# Integrated diagnostics and time series sensitivity assessment for growth monitoring of a medicinal plant (*Glycyrrhiza uralensis* Fisch.) based on unmanned aerial vehicle multispectral sensors

**DOI:** 10.3389/fpls.2025.1612898

**Published:** 2025-08-19

**Authors:** Ao Zhang, Haibin Guan, Zhiheng Dong, Xin Jia, Yan Xue, Fengyu Han, Lingjiang Meng, Xiuling Yu, Xiaoqin Wang, Yang Cao

**Affiliations:** ^1^ College of Pharmacy, Inner Mongolia Medical University, Hohhot, China; ^2^ Department of Pharmacy, Affiliated Hospital of Inner Mongolia Medical University, Hohhot, China; ^3^ Inner Mongolia Tianqi Han and Mongolia Pharmaceutical Co., Ltd., Chifeng, Inner Mongolia Autonomous Region, China

**Keywords:** *Glycyrrhiza uralensis*, machine learning, phenotyping, water management, remote sensing, vegetation indices, nitrogen fertilizer management, yield prediction

## Abstract

**Background:**

Water and nitrogen are essential elements prone to deficiency during plant growth. Current water–fertilizer monitoring technologies are unable to meet the demands of large-scale *Glycyrrhiza uralensis* cultivation. Near-ground remote sensing technology based on unmanned aerial vehicle (UAV) multispectral image is widely used for crop growth monitoring and agricultural management and has proven to be effective for assessing water and nitrogen status. However, integrated models tailored for medicinal plants remain underexplored.

**Methods:**

This study collected UAV multispectral images of *G. uralensis* under various water and nitrogen treatments and extracted vegetation indices (VIs). Field phenotypic indicators (PIs), including plant height (PH), tiller number (TN), soil plant analysis development values (SPAD), and nitrogen content (NC), were synchronously measured. Models were constructed using backpropagation neural network (BP), support vector machine (SVM), and random forest (RF) to evaluate PIs to predict yield and monitor growth dynamics. Yield predictions based on PIs were further compared with validate model performance.

**Results:**

The results demonstrated that both the RF algorithm and excess green index (EXG) exhibited versatility in growth monitoring and yield prediction. PIs collectively achieved high-precision predictions (mean 0.42 ≤ *R^2^* ≤ 0.94), with the prediction of PH using green leaf index (GLI) in BP algorithm attaining peak accuracy (*R²* = 0.94). VIs and PIs exhibited comparable predictive capacity for yield, with multi-indicators integrated modeling significantly enhancing performance: VIs achieved *R²* = 0.87 under RF algorithms, whereas PIs reached *R²* = 0.81 using BP algorithms. Further analysis revealed that PH served as the central predictor, achieving *R²* = 0.74 under standalone predictions of RF algorithm, whereas other parameters primarily enhanced model accuracy through complementarity effects, thereby providing supplementary diagnostic value.

**Conclusions:**

This research established a high-precision, time-efficient, and practical UAV remote sensing–based method for growth monitoring and yield prediction in *G. uralensis*, offering a novel solution for standardized production of medicinal plant resources.

## 1 Introduction

Water and nitrogen serve as the material basis and metabolic components throughout the plant phenologycal cycle (Shi et al., 2024). The dynamics of both are closely related to crop yield and quality and are more prone to deficiency (Sharma et al., 2024). Water serves as a substrate for photosynthesis and the primary transport medium within plants. Water deficiency induces leaf wilting and disrupts assimilate allocation, thereby limiting PH growth and reducing effective tillering capacity (Shahzad et al., 2019). Excessive water induces root hypoxia, suppresses respiratory activity, and inhibits ion uptake, further reducing tiller bud initiation and increasing lodging risk (Okada et al., 2024). Nitrogen is a critical component of proteins, nucleic acids, and chlorophyll, inhibits amino acid synthesis, and accelerates chlorophyll degradation under deficiency conditions; this results in suppressed internode elongation and reduced tiller bud viability (Du et al., 2022). Conversely, excessive nitrogen disrupts carbon–nitrogen metabolism by increasing photorespiratory consumption of assimilates, resulting in excessive tillering and delayed panicle differentiation (Li et al., 2021). Imbalanced water and nitrogen supply exacerbate abnormalities in growth phenotype, ultimately compromising plant stress tolerance and yield qualities (Zentgraf et al., 2025). Therefore, climate-change-triggered frequent occurrence of drought events poses a mounting threat to global agricultural productivity, urgently requiring precision water–nitrogen regulation based on real-time monitoring to safeguard food security and enhance resource use efficiency. The important components of this technology include the precise analysis of water and nutrient dynamics during critical plant growth stages and safeguarding plant growth, development, and physiological activities. Consequently, developing highly precise dynamic growth monitoring systems to deliver accurate water–nutrient regulation strategies constitutes the critical pathway for enhancing crop yield and quality.

*Glycyrrhiza uralensis* Fisch. is a widely used medicinal plant belonging to the Leguminosae family, with a history of medicinal use in China spanning over three millennia (Zhou et al., 2019). Its roots and rhizomes are extensively used across East Asia and South Asia for treating and preventing diseases of respiratory and digestive systems (Lu et al., 2023). China is the largest producer of *G. uralensis* globally, with an annual yield of 100,000–120,000 tons (Yan et al., 2023). The Inner Mongolia region is its genuine production area, where the semiarid climate and sandy soils are optimal for secondary metabolite accumulation in *G. uralensis*. In recent years, overexploitation and environmental degradation have led to the depletion of wild resources, making cultivated *G. uralensis* the primary market source. However, most growers lack scientific water and nutrient management practices, unreasonably replicating staple crop management models during large-scale cultivation. This results in severely compromised yield and quality of cultivated *G. uralensis* characterized by substandard medicinal compound content and diminished herb quality (Wan et al., 2021; Yang et al., 2020), significantly constraining industrial development. Medicinal plants have stringent demands for growth environments and cultivation management strategies because of the specific nature of their applications, necessitating substantial labor inputs. Concurrently, increased urbanization and aging population of China have escalated labor management costs (Volpato et al., 2024). Consequently, under the dual pressures of cultivation scale expansion and rising labor expenses, the development of efficient and reliable decision-support tools for medicinal plant growers has become essential.

During the crop growth period, the requirements for water and fertilizer exhibit dynamic variations due to changes in soil physicochemical properties, temperature fluctuations, and rainfall patterns (Plett et al., 2020). Rational fertilization is critical to ensuring crop yield and quality (Lee et al., 2024). Consequently, dynamic monitoring of water and fertilizer requirements during critical crop growth stages and the timely implementation of supplementary measures are essential. Currently, the primary methods for acquiring growth monitoring parameters include physicochemical analysis in the laboratory, measurements using portable phenotyping instrument, and multispectral technology. Physicochemical analysis in the laboratory relies on the availability of a laboratory environment, entailing laborious and protracted operational procedures (Pun Magar et al., 2025). Additionally, portable phenotyping instruments are constrained to single-plant measurements and exhibit low representativeness (Jiang et al., 2020). Conversely, multispectral technology, founded on the close correlation between crop Phenotypic Indicators (PIs) and spectral characteristics, exhibits notable advantages in terms of rapidity (Yu et al., 2024), nondestructiveness (Itoh et al., 2024), and operational simplicity (Chen et al., 2024a). Although existing proximal multispectral technologies can monitor field conditions with high precision (Roth et al., 2024; Andvaag et al., 2024), the data acquisition efficiency of these systems remain low, unable to meet the real-time monitoring demands of large-scale agricultural fields. With the maturation of unmanned aerial vehicle (UAV) technology, platforms can be flexibly equipped with payloads such as Red-Green-Blue (RGB) sensors, multispectral sensors, hyperspectral sensors, thermal infrared sensors, and Light detection and ranging (LiDAR) (Zhang et al., 2024). This enables efficient capture of photon scattering, absorption, and transmission processes resulting from light–plant–canopy interactions. Through radiative transfer modeling, the light attenuation effects associated with vegetation physicochemical attributes are quantified, thereby precisely resolving crop PIs. These capabilities facilitate growth dynamic monitoring (Sumnall et al., 2024), nutritional status diagnosis (Zhu et al., 2024), and yield prediction (Shen et al., 2024). Currently, UAV-based low-altitude spectral imaging technology provides an innovative technical pathway for growth monitoring in large-scale *G. uralensis* cultivation zones, leveraging its efficiency, real-time performance, and nondestructive advantages. This framework extends to staple crop surveillance, delivering cost-effective and highly adaptable smart agricultural solutions for global food security.

The process of crop growth detection must take consider agroecosystem sustainability. This technological challenge is addressed through UAV multispectral technology, which provides nondestructive, low-cost, and efficient monitoring capabilities. Multispectral sensors can be equipped with multiple discrete spectral bands. The selection of these bands is determined by the necessity of vegetation indices (VIs), which are correlated with crop phenotypes. These indices demonstrate higher sensitivity to vegetation characteristics compared with single-wavelength data. Among them, VIs including the normalized difference vegetation index (NDVI), green normalized difference vegetation index (GNDVI), normalized difference red-edge index (NDRE), and soil-adjusted vegetation index (SAVI), have been demonstrated to strongly correlate with plant nutritional status (Song et al., 2022). Duque et al. (2023) employed a combination of GNDVI and SAVI, along with phenotypic data and Gaussian regression, to estimate nitrogen levels in rice with high precision. In a similar study, Ali et al. (2024) correlated soil plant analysis development (SPAD) values from sugar beet parts with NDVI and developed an SVM-based model for accurate nitrogen diagnosis and drought response. Zou et al. (2024) developed a UAV-based leaf area index (LAI) estimation framework integrating spectral indices, optimized texture features, and plant height (PH) through machine algorithms, demonstrating enhanced robustness against spectral saturation through multifeature fusion. Johansen et al. (2020) developed models to predict tomato biomass and yield by integrating plant morphological and spectral features extracted from UAV-based RGB and multispectral imagery with the random forest (RF) and further investigated prediction discrepancies under salt-stress conditions. Based on the aforementioned evidence, we speculated that UAV multispectral technology has significant potential for establishing growth monitoring models for *G. uralensis*, addressing urgent market demands for cultivated varieties that balance high yield with superior quality.

Machine learning algorithms enhance model performance by effectively capturing complex data relationships. backpropagation neural network (BP) excels at learning intricate nonlinear patterns but requires substantial data and computational resources and is prone to over-fitting. SVM is theoretically grounded and exhibits strong generalization capabilities with small-sample, high-dimensional data. However, its effectiveness highly depends on kernel function and parameter selection, and it offers limited interpretability for nonlinear relationships. RF is user-friendly, achieves high accuracy, demonstrates high resistance to overfitting, has flexible data requirements, provides feature importance rankings, and supports parallel computation but exhibits limited extrapolation capability. Considering these distinct algorithmic characteristics, the split ratio between training and test sets is crucial. It must balance model learning capacity with robust generalization validation: sufficient training data enable effective learning and mitigate underfitting, whereas an adequately sized test set ensures reliable and statistically significant performance evaluation. Therefore, for UAV-based monitoring and yield prediction of *G. uralensis*, in-depth understanding and optimized application of these algorithms are crucial for enhancing the efficacy of technology.

The objective of this study was to validate the feasibility of real-time and efficient growth monitoring of a medicinal plant *G. uralensis* using UAV multispectral technology. Moreover, the study further evaluated the accuracy of different algorithms to predict PIs and established a PIs-based yield prediction model. It is hypothesized that the integration of UAV multispectral technology with optimized algorithms will facilitate high-precision growth monitoring and yield prediction based on PIs.

## 2 Materials and methods

### 2.1 Experimental design and data collection

#### 2.1.1 Study area and experimental design

**Figure 1:**
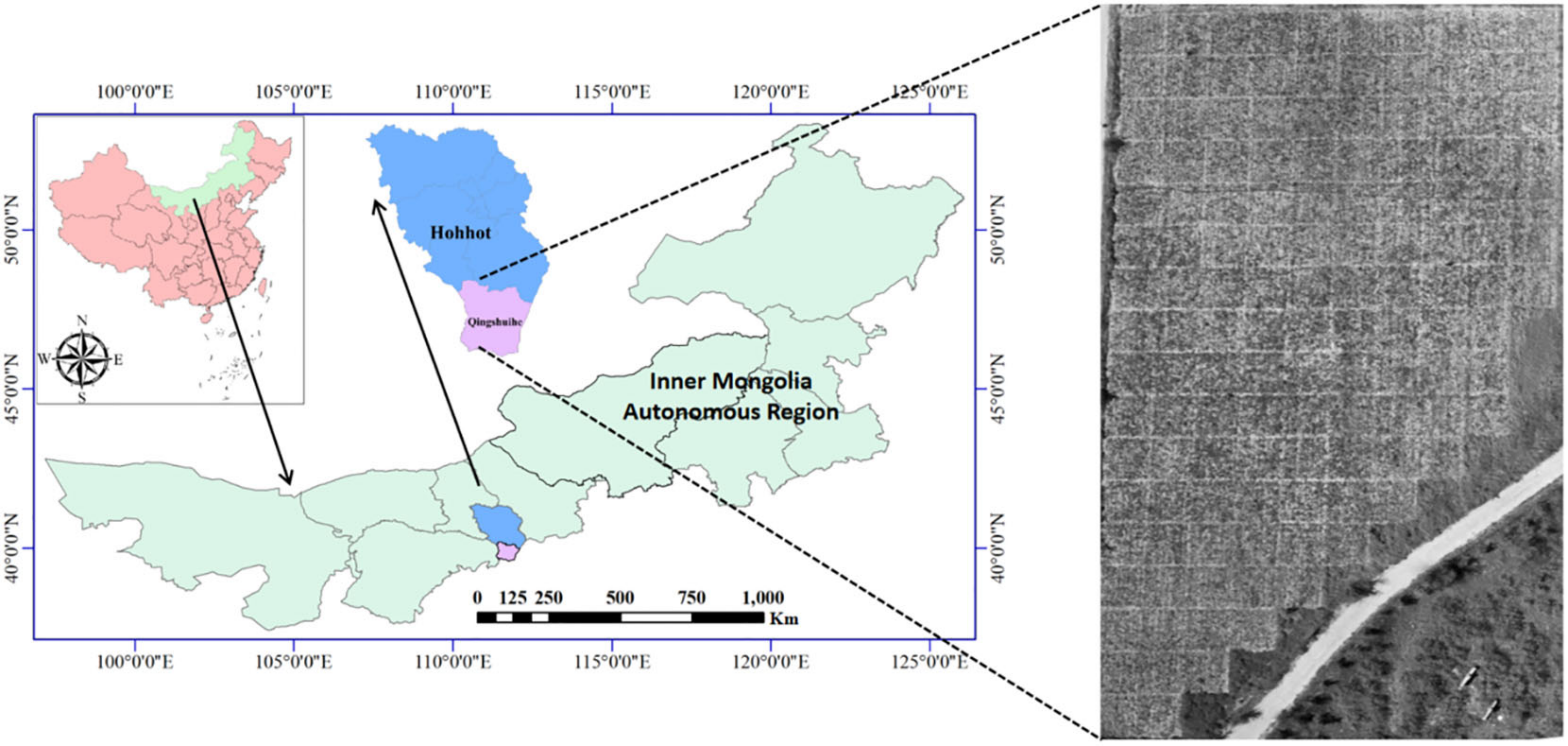
Experimental region and design of different water and fertilizer treatments.

The field experiment was conducted in Qingshuihe County (41°8′ N, 112°10′ E; 1,100 m ASL), Hohhot City, Inner Mongolia Autonomous Region, China, from June 2022 to September 2023 (**Figure 1**). This area lies within the extended zone of the *G. uralensis* genuine producing region on the Ordos Plateau, characterized by a temperate continental monsoon climate. The locally arid conditions favor the synthesis and accumulation of secondary metabolites in *G. uralensis*. During the experimental period, the recorded average diurnal temperature was 13.60°C; average nocturnal temperature was 2.25°C, and cumulative rainfall reached 413.80 mm. The soil type is clay loam (pH 8.0), supporting natural distribution of wild *G. uralensis* and demonstrating suitability for cultivated research. Based on the growth cycle from sowing in mid-May to harvesting in early October, the period was divided into four important phenological stages: seedling establishment [40 DAS (days after sowing)], rapid vegetative growth (85 DAS), reproductive-storage transition (110 DAS), and maturation and harvest (145 DAS).

The experiment was performed in a regional mountainous environment with four irrigation levels (2,000, 4,000, 6,000, and 8,000 m³/ha) and five nitrogen application levels (75, 150, 225, 300, and 375 kg N/ha). The control treatment (CK) included neither supplemental irrigation nor fertilization. Each treatment was replicated three times (63 experimental plots in total; 5 m × 5 m per plot), with 1 m wide alleys separating adjacent plots. Compound fertilizer (N:P_2_O_5_:K_2_O = 20:20:20) was applied as base fertilizer. Urea was used for nitrogen treatment. Fertilizers were uniformly incorporated into the soil layer during plowing. Post-sowing irrigation was supplied using a drip system. Data were collected 30 days after sowing and thereafter at monthly intervals. All measurements were conducted under clear, windless conditions (10:00–15:00) to minimize temporal discrepancies between ground observations and UAV remote sensing.

#### 2.1.2 Remote sensing image acquisition

Multispectral image data were acquired during phenological stages phenological stages (seedling establishment, rapid vegetative growth, reproductive-storage transition, and maturation and harvest). Image collection was uniformly conducted between 11:00 and 13:00 under clear and calm atmospheric conditions. A DJI Phantom 4 Multispectral UAV (DJI Inc., Shenzhen, China) equipped with a multispectral camera system was deployed (**Supplementary Table S1**), featuring six CMOS sensors (Sony Group Corporation, Minato, Japan), one color sensor for visible-light imaging and five monochromatic sensors for multispectral imaging (**Supplementary Table S2**). Flight missions were programmed and executed via the DJI GS Pro software (DJI Inc., Shenzhen, China) running on an external tablet device (iPad mini, Apple Inc., USA) mounted to the drone remote controller. Routes were planned using GPS positioning and 2D map clipping. The platform operated at 50 m above ground level with a horizontal speed of 5.0 m/s, ensuring 2.6 cm/pixel spatial resolution. A gimbal-stabilized camera maintained a nadir orientation relative to the terrain surface. Image collection followed predefined flight routes with 80% longitudinal overlap and 75% lateral overlap between consecutive frames. Before data acquisition, three radiometric calibration panels (25%, 50%, 75% reflectance values; JINGYI, Guangzhou, China) were deployed at plot centers to validate radiometric calibration integrity.

#### 2.1.3 Ground data collection

Ground data collection was synchronized with UAV multispectral remote sensing data acquisition. The TYS-4N device (TOP Cloud-agri, Hangzhou, China) was used to measure SPAD and nitrogen content (NC). Measurements were conducted on the first fully expanded leaf beneath the terminal branch, with five positions sampled: both lateral sides of the leaf base, both lateral sides of the mid-section, and the leaf tip; the mean value of these points was calculated. PH was manually recorded by extending a 1-m ruler vertically from the base to the apex of the main stem. Yield was obtained through destructive sampling by harvesting entire plants from the soil, and tiller number (TN) per plant was manually determined at the late tillering stage by counting all tillers with at least two visible leaves. Each treatment was randomly sampled in five replicates.

### 2.2 Digital image processing and data analysis

#### 2.2.1 Generation of orthorectified mosaic and radiometric correction

Multispectral UAV image processing was implemented in Pix4Dmapper version 4.3.9 (Pix4D SA, Lausanne, Switzerland). The workflow encompassed image alignment, 3D terrain reconstruction, lens aberration correction, and dark angle compensation, followed by core radiometric calibration:


$$X_{ref}=\frac{X_{DN}\times pCam_{X}}{X_{LS}\times pLS_{X}}\times \rho_{NIR}$$

Where *X_DN_* denotes the brightness value of the image element; *X_LS_* represents light-sensitive signal captured in real-time by the light intensity sensor; *pCam_x_* and *pLS_x_* are the calibration parameters for the multispectral camera and light sensor, respectively, and *ρ_NIR_* serves as the parameter regulating interconversion referenced to the near-infrared band. This algorithm eliminates ambient light fluctuations through dynamic irradiance monitoring while standardizing sensor responses via integrated hardware calibration parameters.

#### 2.2.2 Multispectral image stitching and extraction of VIs

**Figure 2:**
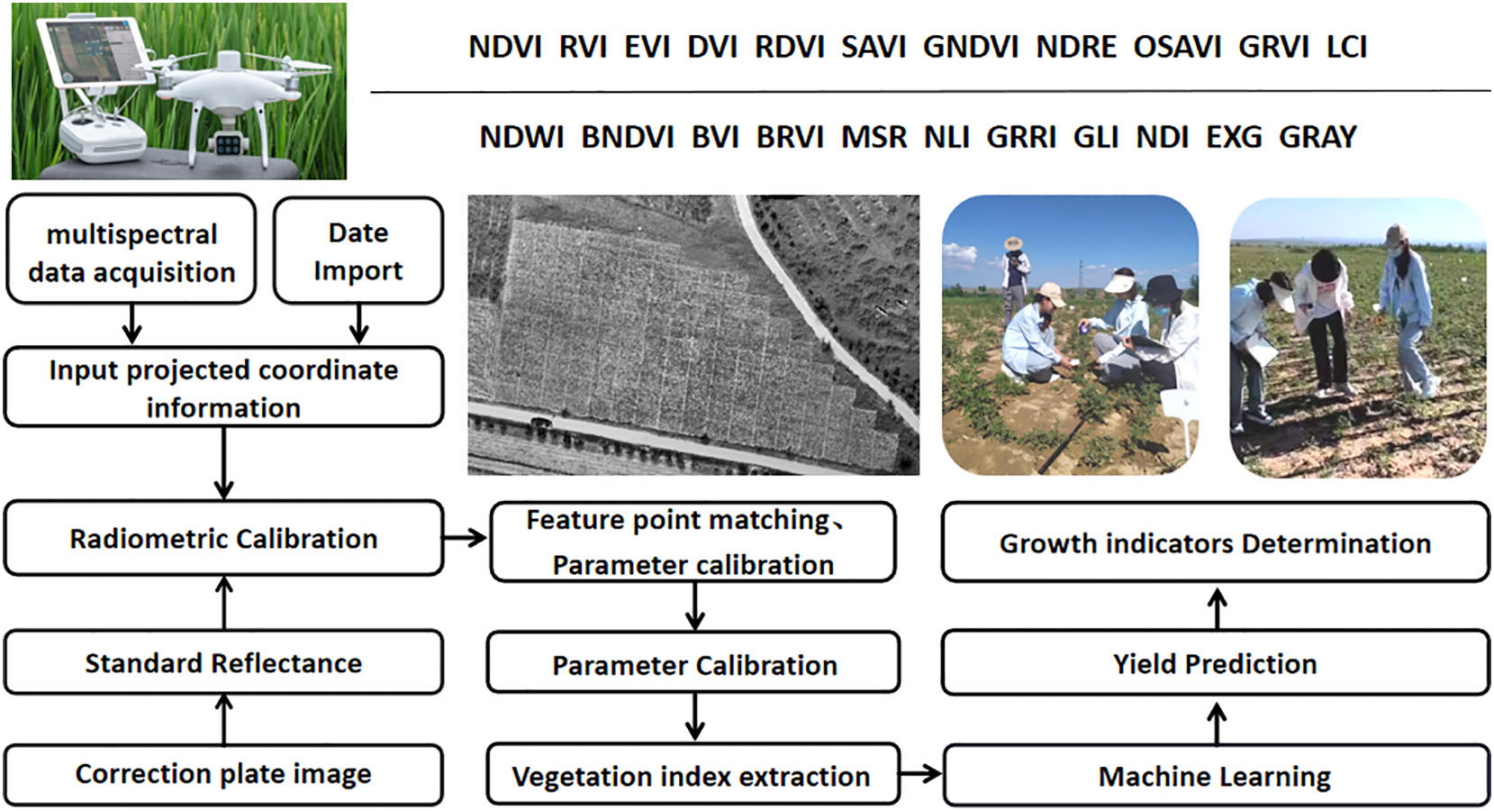
Multispectral data processing flow of UAV.

The captured RGB images underwent geometric correction via orthorectification and were subsequently mosaicked in Pix4Dmapper (**Figure 2**). Orthorectification corrected spatial distortions caused by differences in terrain elevation and camera tilt angles to ensure geometric accuracy. High overlap rates (80% longitudinal, 75% lateral) enhanced redundant image matching during stitching, effectively reducing gaps and positional errors in the composite. These steps significantly minimized seamline artifacts inherent to mosaicking. All processing used photogrammetric tools of Pix4Dmapper. Finally, structure-from-motion (SFM) algorithms generated digital surface models (DSM) and georeferenced orthophotos. VIs were calculated in Pix4Dmapper based on spectral data from the processing area (**Table 1**).

**Table 1:** Vegetation indices and equations.

Name	Equation	Effect	Reference
Normalized Difference VI	NDVI=(NIR–R)/(NIR+R)	Vegetation density and health estimation	(Filonchyk et al., 2025)
Red Ratio VI	RVI=NIR/R	Vegetation vigor quantification	(Li et al., 2025a)
Enhanced VI	EVI=2.5*((NIR-R)/(NIR+6R-7.5B+1))	High-biomass sensitivity enhancement	(Tang et al., 2025)
Difference VI	DVI=NIR-R	Biomass estimation via NIR - red difference	(Tang et al., 2025)
Renormalized Difference VI	RDVI=(NIR-R)/√(NIR-R)	Soil background noise minimization	(Tang et al., 2025)
Soil Adjusted VI	SAVI=1.5*(NIR-R)/(NIR+R+0.5)	Soil brightness variation compensation	(Yueliang et al., 2025)
Green Normalized Difference VI	GNDVI=(NIR-G)/(NIR+G)	Chlorophyll sensitivity enhancement	(Basso et al., 2019)
Normalized Difference Red-edge VI	NDRE=(NIR–RE)/(NIR+RE)	Chlorophyll content change detection	(Li et al., 2025a)
Optimization of Soil-Adjusted VI	OSAVI=(NIR-R)/(NIR+R+0.16)	Optimized soil-adjusted vegetation index	(Ashrafuzzaman et al., 2025)
Green Ratio VI	GRVI=NIR/G	Early growth stage sensitivity	(Sapkota and Paudyal, 2023)
Leaf Chlorophyll Index	LCI=(NIR-RE)/(NIR-R)	Chlorophyll density targeting	(Yu et al., 2022)
Normalized Difference Water Index	NDWI=(G-NIR)/(G+NIR)	Plant water content assessment	(Jurevičius et al., 2022)
Blue Normalized Difference VI	BNDVI=(NIR-B)/(NIR+B)	Atmospheric scattering reduction	(Traba et al., 2022)
Blue Ratio VI	BVI=NIR/B	Early stress detection	(Liao et al., 2025)
Simple Blue Ratio Index	BRVI=R/B	Soil contrast enhancement	(Liao et al., 2025)
Modified Simple Ratio	MSR=(NIR/R-1)/√(NIR/B+1)	High-biomass saturation mitigation	(Wang et al., 2022)
Non-Linear Index	NLI=(NIR*NIR-R)/(NIR*NIR+R)	LAI estimation via non-linear transformation	(Huang et al., 2025)
Green-Red Ratio Index	GRRI=R/G	Senescence detection	(Xu et al., 2022)
Green Leaf Index	GLI=(2*G-R-B)/(2*G+R+B)	General vegetation health assessment	(Li et al., 2025b)
Normalized Difference Index	NDI=(R-G)/(R+G+0.01)	Custom band combination framework	(Deveerasetty et al., 2024)
Excess Green Index	EXG=2*G-B-R	Green vegetation highlighting	(Zanotta et al., 2025)
Gray Level VI	GRAY=(R+G+B)/3	Soil brightness measurement; Background noise correction	(Hu et al., 2024)

#### 2.2.3 Data analysis

A simple linear regression analysis was performed on UAV-based VIs and manually measured PI data to explore their relationship. In this model, the field-acquired PIs served as the dependent variables, whereas the sensor-derived VIs served as the independent predictors. Validation involved comparing VI-estimated PIs against ground-truth measurements. Estimation accuracy was quantified using four metrics: coefficient of determination (*R²*), root mean square error (*RMSE*), mean absolute error (*MAE*), and mean bias error (*MBE*). *R²* quantified the proportion of variance explained by the model, with higher values indicating stronger correlations. *RMSE* and *MAE* represented the magnitude of prediction errors, with lower values reflecting higher precision. *MBE* indicated systematic overestimation or underestimation tendencies, with values closer to zero representing minimal bias. Mathematical formulae of these metrics were defined to ensure transparent interpretation of model performance.


$$R^{2}=1-\frac{\sum_{i=1}^{n}{\left(y_{i}-{\hat{y}}_{i}\right)}^{2}}{\sum_{i=1}^{n}{\left(y_{i}-{\bar{y}}_{i}\right)}^{2}}$$

$$RMSE=\sqrt{\frac{1}{n}\sum_{i=1}^{n}{\left({\hat{y}}_{i}-y_{i}\right)}^{2}}$$

$$MAE=\frac{1}{n}\sum_{i=1}^{n}\left|{\hat{y}}_{i-}y_{i}\right|$$

$$MBE=\frac{1}{n}\sum_{i=1}^{n}\left|{\hat{y}}_{i}-y_{i}\right|$$

Where n is the number of samples; *yi* and ${\hat{y}}_{i}$ are the measured and estimated values, respectively; $\bar{y}$ i is average measured value.

#### 2.2.4 Model construction

Based on the Pearson correlation coefficient, the top three VIs significantly correlating with each PI were defined as the independent variables for the model. Three linear machine learning algorithms, specifically the BP, SVM, and RF, were used to model relationships between VIs and PIs. The dataset was randomly partitioned at the plot level into training (70%) and testing (30%) subsets, with strict isolation between the two groups to prevent data leakage. Model parameters were optimized using the training subset, whereas the testing subset independently evaluated predictive performance. Implemented via MATLAB (MathWorks R2019b Inc., Natick, MA, USA)-customized linear techniques, each algorithm (BP, SVM, and RF) underwent iterative training on the training data. Model accuracy was assessed using *R²*, *RMSE*, *MAE*, and *MBE* metrics to quantify agreement between predicted and observed PIs.

## 3 Results

### 3.1 Effect of water and fertilizer treatments on the growth of *G. uralensis*

Samples were collected after 2 years of water and fertilizer treatments and the yield, PH, leaf chlorophyll content, and NC of *G. uralensis* across different water treatment groups (**Table 2**) were measured. Except W2, all water-stress treatment groups exhibited significant decreases in PH compared with CK, with W3 exhibiting the highest decrease (11.39%), followed by W1 (10.30%), and W4 exhibited the smallest decrease (4.69%). SPAD exhibited significant differences only between W2 and CK (9.44% decrease). W3 displayed significant increase in SPAD compared with CK (2.57%), with no statistical differences observed in other groups. No significant differences were observed in TN among water treatment groups (*P* > 0.05). For NC, no significant differences were detected between any treatment group and CK, although W3 exhibited 4.40% increase in NC compared with CK, indicating potential for water regulation. Collectively, all water stress treatments decreased PH (W3 > W1 > W4 > W2). Only W2 significantly decreased photosynthetic pigment content (SPAD), whereas W3 exhibited coordinated improvements in SPAD and NC despite decreased PH.

**Table 2:** The effect of different water treatments on phenotypic indicators.

Group	PH/cm	TN	SPAD	NC/mg*g^-1^
CK	53.58±0.05a	1.67±0.58a	41.62±0.05ab	3.41±0.10a
W1	48.07±3.89b	1.47±0.51a	41.01±2.87ab	3.43±0.24a
W2	52.13±2.87a	1.33±0.51a	37.69±2.52b	3.36±0.20a
W3	47.47±2.77b	1.47±0.49a	42.69±4.03a	3.56±0.34a
W4	51.07±4.31ab	1.67±0.49a	40.34±3.72ab	3.49±0.32a

LSD, Different lowercase letters on the table indicate significant differences between treatments (*P* < 0.05). PH stand for plant height; TN stand for tiller number; SPAD stand for soil and plant analysis development value; NC stand for nitrogen content.

Samples were collected after 2 years of water and fertilizer treatments, and the yield, PH, leaf chlorophyll content, and NC of *G. uralensis* across different fertilizer treatment groups (**Table 3**) were measured. Compared with the CK group, the N1, N2, and N5 groups exhibited significant decreases in PH (9.17%–10.41%), whereas the N3 and N4 groups exhibited no significant differences in PH compared with CK (reductions of 5.28%–1.70%). SPAD exhibited significant decrease of 11.63% and 9.80% in the N1 and N2 groups, respectively, with no statistical differences observed in the N3, N4, or N5 groups. NC was significantly higher in the N4 and N5 groups than in the CK group (increases of 7.33% and 1.47%, respectively), whereas no significant differences were observed in the remaining treatment groups. Tillering numbers exhibited no significant difference among all nitrogen treatment groups (*P* > 0.05). Collectively, nitrogen treatments induced differential responses: N1 and N2 caused marked decline in PH and chlorophyll levels; N4 and N5 enhanced nitrogen accumulation, and no treatment significantly affected tillering capacity.

**Table 3:** The effect of different nitrogen treatments on phenotypic indicators.

Group	PH/cm	TN	SPAD	NC/mg*g^-1^
CK	53.58±1.58a	1.67±0.58a	41.62±0.05ab	3.41±0.10a
N1	48.67±3.74b	1.58±0.51a	36.78±2.16b	3.23±0.18b
N2	48.33±4.25b	1.58±0.51a	39.99±3.79ab	3.43±0.29ab
N3	50.75±3.73ab	1.25±0.49a	40.78±3.34a	3.53±0.27ab
N4	52.67±4.56ab	1.33±0.51a	42.27±5.00a	3.66±0.29a
N5	48.00±2.10b	1.33±0.49a	42.34±4.33a	3.46±0.39a

LSD, Different lowercase letters on the table indicate significant differences between treatments (*P* < 0.05). PH stand for plant height; TN stand for tiller number; SPAD stand for soil and plant analysis development value; NC stand for nitrogen content.

### 3.2 Model building and evaluation

#### 3.2.1 Variable filtering

**Figure 3:**
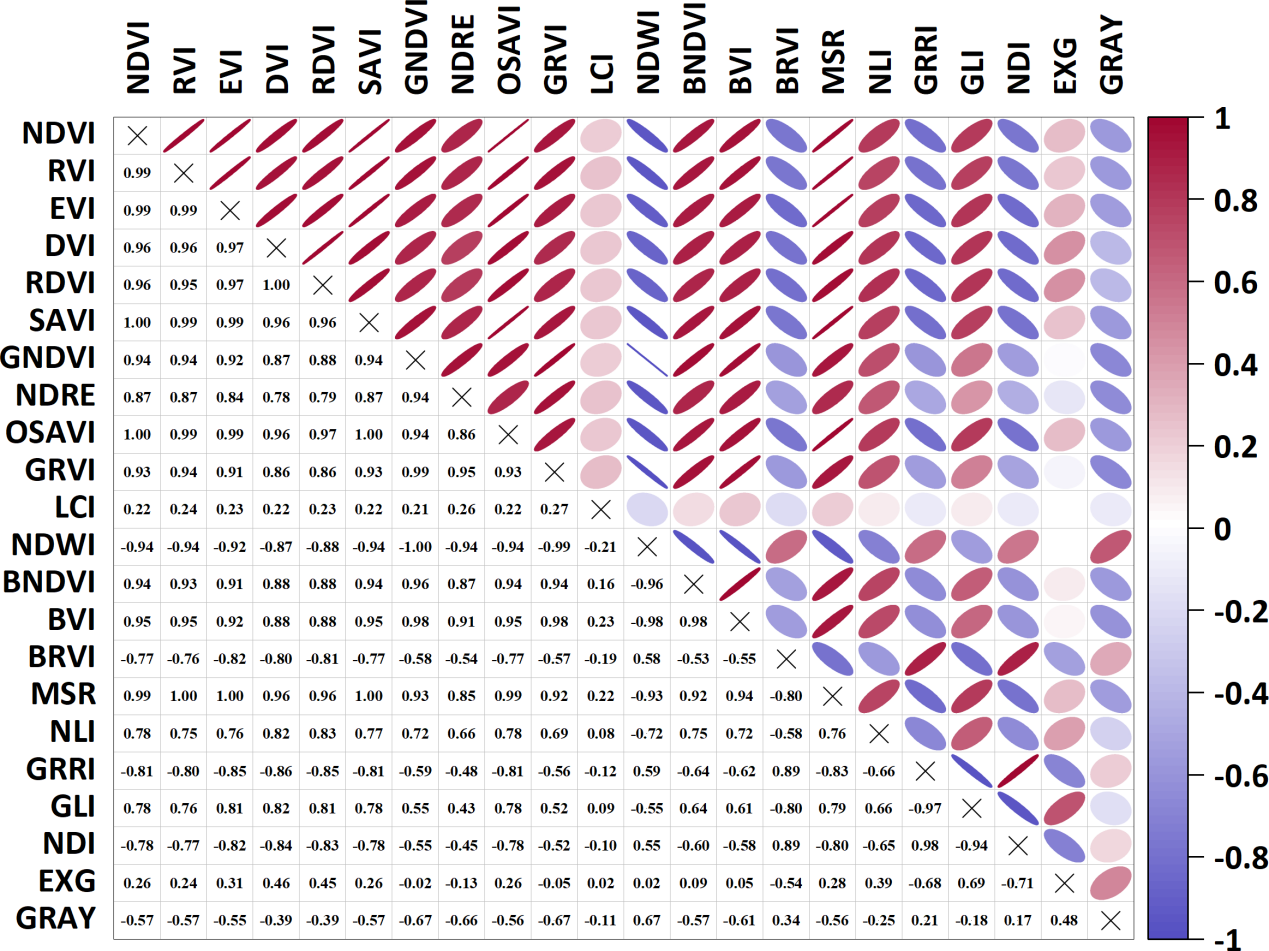
Heat map of vegetation indices correlation.

**Figure 4:**
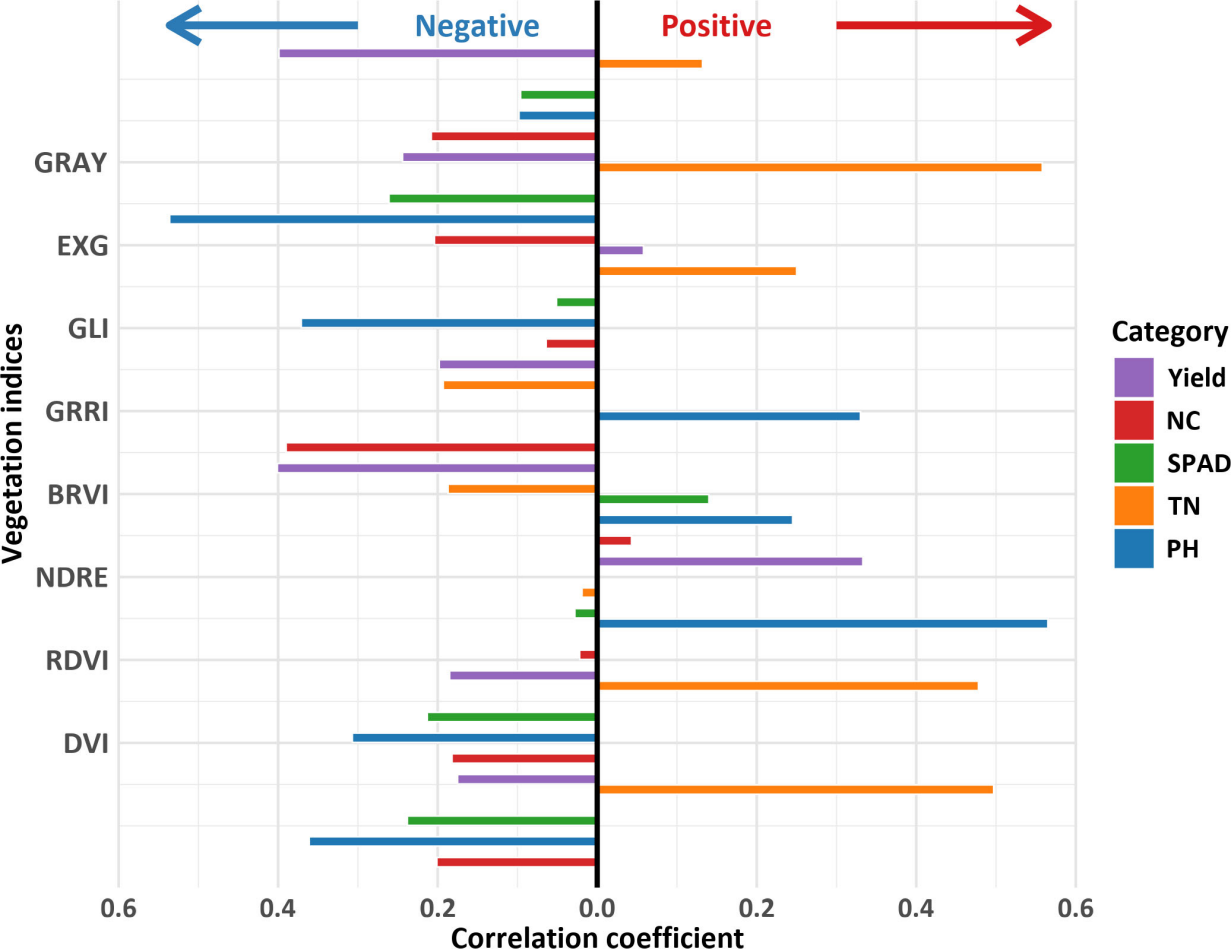
Correlation between phenotypic indicators and vegetation indices. PH stand for plant height; TN stand for tiller number; SPAD stand for soil and plant analysis development value; NC stand for nitrogen content. GRAY stand for gray level index; EXG stand for excess green index; GLI stand for green leaf index; GRRI stand for green - red ratio index; BRVI stand for simple blue ratio index; NDRE stand for normalized difference red - edge index; RDVI stand for renormalized difference vegetation index; DVI stand for difference vegetation index.

Pearson correlation analysis was performed to assess pairwise correlations among 22 VIs for redundancy elimination (**Figure 3**). NDVI, EVI, SAVI, OSAVI, MSR, and RVI exhibited marked redundancy (*r* ≥ 0.99), indicating that these indices should be excluded from subsequent modeling. Subsequently, VIs were screened based on yield and PIs to select model inputs (**Figure 4**). Prediction models for PIs and yield were developed using BP, SVM, and RF algorithms with the optimized VI sets. A standalone model directly linking PIs to yield was concurrently established to enable comparative evaluation, which validated the synergistic effects of the multidimensional modeling framework.

#### 3.2.2 Estimation of PIs based on VIs

**Figure 5:**
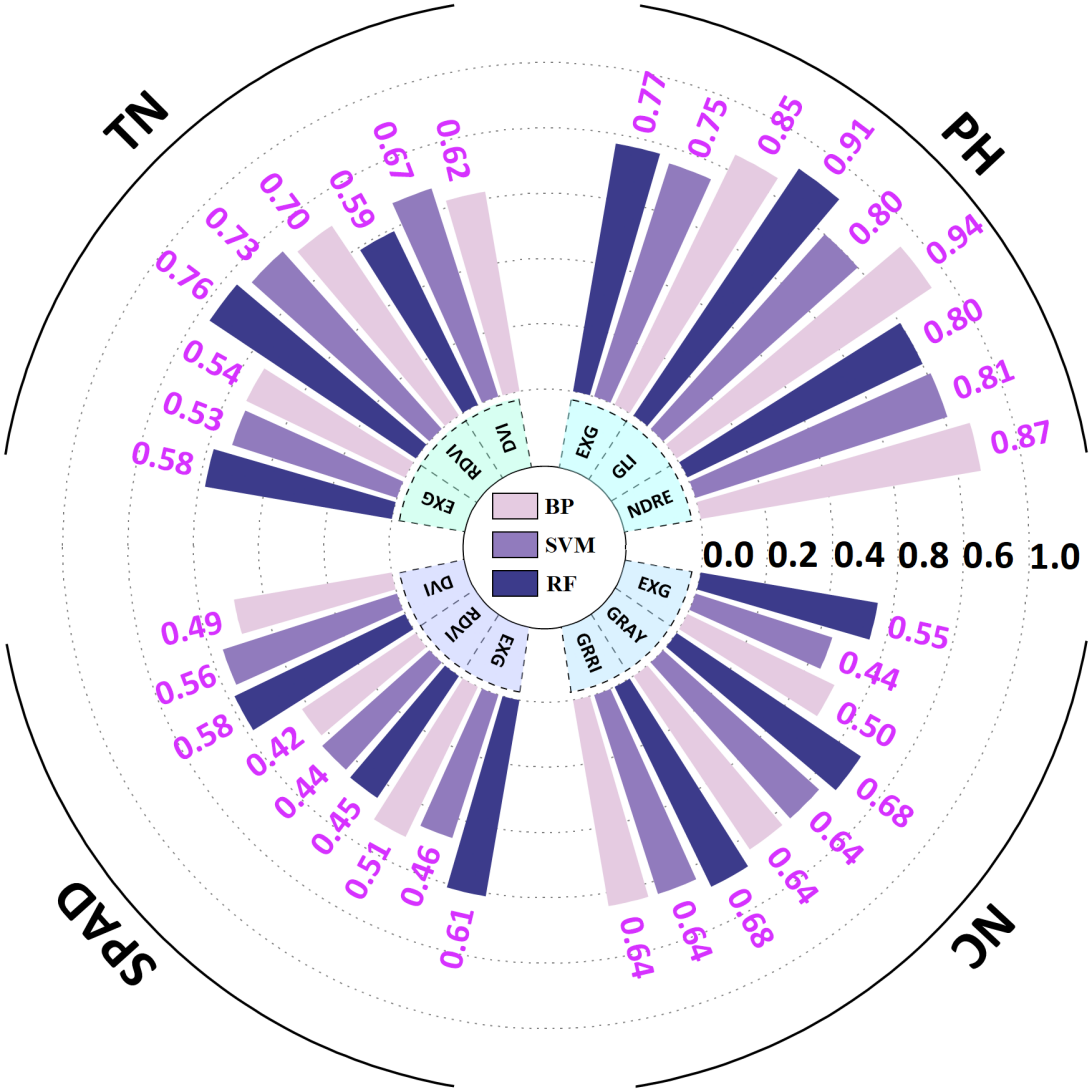
Radial bar chart of *G. uralensis* phenotypic indicator prediction results based on vegetation indices. PH stand for plant height; TN stand for tiller number; SPAD stand for soil and plant analysis development value; NC stand for nitrogen content. EXG stand for excess green index; GLI stand for green leaf index; NDRE stand for normalized difference red - edge index; GRAY stand for gray level index; GRRI stand for green - red ratio index; RDVI stand for renormalized difference vegetation index; DVI stand for difference vegetation index. BP stand for back propagation neural network; SVM stand for support vector machine; RF stand for random forest.

The VI-based phenotypic prediction model for *G. uralensis* (**Table 4**) exhibited significant correlations between predicted and measured values of SPAD, NC, TN, and PH (*R²* > 0.40). GLI, excess green index (EXG), and DVI under the BP algorithm exhibited high accuracy in predicting PH (*R²* > 0.85), with GLI predicting the best among the three VIs, and the BP algorithm having the highest prediction accuracy (*R²* = 0.94, *RMSE* = 2.70). RF algorithm also exhibited high prediction accuracy (*R²* = 0.91, *RMSE* = 2.87). The RF models of GRRI and GRAY exhibited the highest prediction accuracy for NC (*R²* = 0.68, *RMSE* = 0.01 and 0.08), and the combination of RF and RDVI best predicted TN (*R²* = 0.76, *RMSE* = 0.21; **Figure 5**). Scatter plots and bar charts of the prediction results are given in **Supplementary Figures S1** and **S2**. The RF algorithm, which is based on the advantages of integrated analysis of morphological and physiological characteristics, exhibited stronger generalization ability in predicting different PIs, whereas EXG exhibited higher accuracy in the prediction model, making it a critical spectral discriminant for monitoring the growth of *G. uralensis*.

**Table 4:** VIs prediction phenotype indictors results.

Phenotypic indicators	VIs	Algorithm	R^2^	RMSE	MAE	MBE	Equation
SPAD	DVI	BP	0.49	1.33	1.67	0.23	y=0.53x+19.04
		SVM	0.56	1.27	1.67	0.24	y=0.51x+19.72
		RF	0.58	1.08	1.49	-0.05	y=0.52x+19.42
	RDVI	BP	0.42	1.79	1.59	-0.06	y=0.53x+18.45
		SVM	0.44	1.92	3.82	-0.98	y=0.51x+14.95
		RF	0.45	0.44	5.19	2.41	y=0.52x+12.49
	EXG	BP	0.51	1.66	1.71	-0.16	y=0.52x+18.98
		SVM	0.46	1.42	1.81	-0.26	y-0.38x+24.45
		RF	0.61	1.36	1.43	-0.05	y=0.55x+17.94
NC/mg*g^-1^	GRRI	BP	0.64	0.09	0.13	0.17	y=0.41x+1.97
		SVM	0.64	0.07	0.14	-0.01	y=0.43x+1.90
		RF	0.68	0.01	0.13	0.00	y=0.48x+1.76
	GRAY	BP	0.64	0.09	2.59	-3.52	y=0.41x+0.97
		SVM	0.64	0.07	5.85	-3.59	y=0.39x+10.86
		RF	0.68	0.08	3.35	0.02	y=0.49x+1.68
	EXG	BP	0.50	0.16	0.12	0.16	y=0.41x+1.93
		SVM	0.44	0.16	0.13	-0.01	y=0.42x+1.90
		RF	0.55	0.16	0.12	0.00	y=0.47x+1.76
TN	DVI	BP	0.62	0.22	0.18	0.02	y=0.91x+0.17
		SVM	0.67	0.29	0.24	-0.03	y=0.73x+0.43
		RF	0.59	0.25	0.19	0.00	y=0.84x+0.29
	RDVI	BP	0.70	0.24	0.18	-0.01	y=0.93x+0.10
		SVM	0.73	0.24	0.20	0.02	y=0.81x+0.35
		RF	0.76	0.21	0.18	-0.01	y=0.83x+0.30
	EXG	BP	0.54	0.23	0.19	-0.03	y=0.89x+0.17
		SVM	0.53	0.23	0.22	-0.04	y=0.77x+0.37
		RF	0.58	0.21	0.17	0.00	y=0.86x+0.23
PH/cm	NDRE	BP	0.87	3.51	3.08	0.03	y=0.85x+6.53
		SVM	0.81	3.86	3.76	0.00	y=0.77x+10.01
		RF	0.80	2.77	2.74	0.10	y=0.80x+8.72
	GLI	BP	0.94	2.70	2.26	0.36	y=0.88x+5.46
		SVM	0.80	3.79	3.44	-1.09	y=0.80x+7.40
		RF	0.91	2.87	2.83	0.14	y=0.78x+9.53
	EXG	BP	0.85	3.29	2.60	-0.07	y=0.85x+6.39
		SVM	0.75	3.91	4.01	-0.38	y=0.68x+13.12
		RF	0.77	3.74	3.35	0.02	y=0.74x+10.66

PH stand for plant height; TN stand for tiller number; SPAD stand for soil and plant analysis development value; NC stand for nitrogen content. EXG stand for excess green index; GLI stand for green leaf index; NDRE stand for normalized difference red - edge index; GRAY stand for gray level index; GRRI stand for green - red ratio index; RDVI stand for renormalized difference vegetation index; DVI stand for difference vegetation index. BP stand for back propagation neural network; SVM stand for support vector machine; RF stand for random forest.

#### 3.2.3 Yield prediction based on VIs

**Figure 6:**
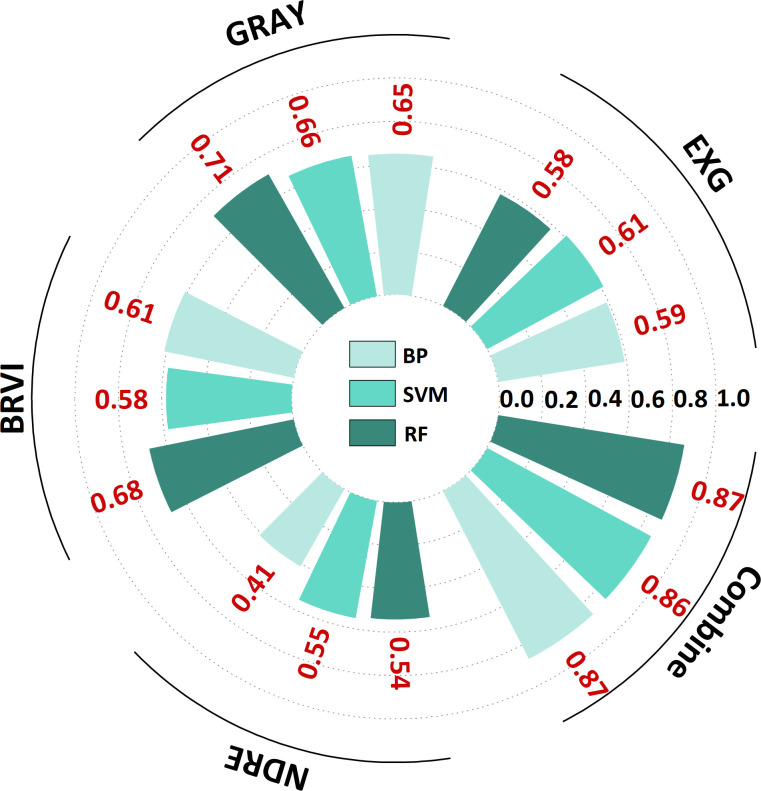
Radial bar chart of *G. uralensis* yield prediction based on vegetation indices. EXG stand for excess green index; GLI stand for green leaf index; GRAY stand for gray level index; BRVI stand for simple blue ratio index; NDRE stand for normalized difference red - edge index. BP stand for back propagation neural network; SVM stand for support vector machine; RF stand for random forest.

The findings of the yield prediction study (**Table 5**) demonstrated that BRVI, EXG, GRAY, and NDRE substantially correlated with *G. uralensis* yield (*R²* > 0.40). Furthermore, the prediction accuracy of the multi-index combined model was significantly superior to that of a single-index. Specifically, EXG and NDRE demonstrated optimal performance in the SVM algorithm (*R²* = 0.61, *RMSE* = 15.53; *R²* = 0.55, *RMSE* = 13.27), whereas GRAY and BRVI achieved the highest accuracy through the RF algorithm (*R²* = 0.71, *RMSE* = 10.94; *R²* = 0.68, *RMSE* = 11.37). In the multi-index fusion model, a high-accuracy prediction model was achieved by the BP algorithm (*R²* = 0.87, *RMSE* = 10.67), and the RF algorithm further reduced the *RMSE* to 6.78 (**Figure 6**). Scatter plots and bar charts of the prediction results are given in **Supplementary Figure S3** and **S4**. The RF algorithm demonstrated a high integrated prediction ability among the three algorithms, both for single-index and multi-index combined predictions, which fully validated its stability and high efficiency in yield prediction.

**Table 5:** VIs predicts yield results.

VIs	Algorithm	R^2^	RMSE	MAE	MBE	Equation
EXG	BP	0.59	11.02	14.61	-0.18	y=0.40x+38.51
	SVM	0.61	15.43	13.32	1.11	y=0.67x+23.80
	RF	0.58	10.78	11.00	-0.02	y=0.54x+29.02
GRAY	BP	0.65	12.27	12.41	-0.32	y=0.59x+25.86
	SVM	0.66	14.65	9.65	2.27	y=0.77x+17.89
	RF	0.71	10.94	8.25	0.21	y=0.69x+19.57
BRVI	BP	0.61	11.82	11.06	0.07	y=0.61x+25.62
	SVM	0.58	11.07	9.93	-3.15	y=0.52x+27.34
	RF	0.68	11.37	8.74	0.28	y=0.69x+20.50
NDRE	BP	0.41	13.17	11.83	0.48	y=0.47x+34.39
	SVM	0.55	13.27	13.37	-0.80	y=0.45x+33.99
	RF	0.54	11.45	11.52	0.07	y=0.47x+33.85
Combine	BP	0.87	10.67	7.25	-2.05	y=0.83x+8.75
	SVM	0.86	10.75	5.93	-1.05	y=0.83x+9.85
	RF	0.87	6.78	5.63	-0.03	y=0.8x+12.46

EXG stand for excess green index; GLI stand for green leaf index; GRAY stand for gray level index; BRVI stand for simple blue ratio index; NDRE stand for normalized difference red - edge index. BP stand for back propagation neural network; SVM stand for support vector machine; RF stand for random forest.

#### 3.2.4 Yield prediction based on PIs

**Figure 7:**
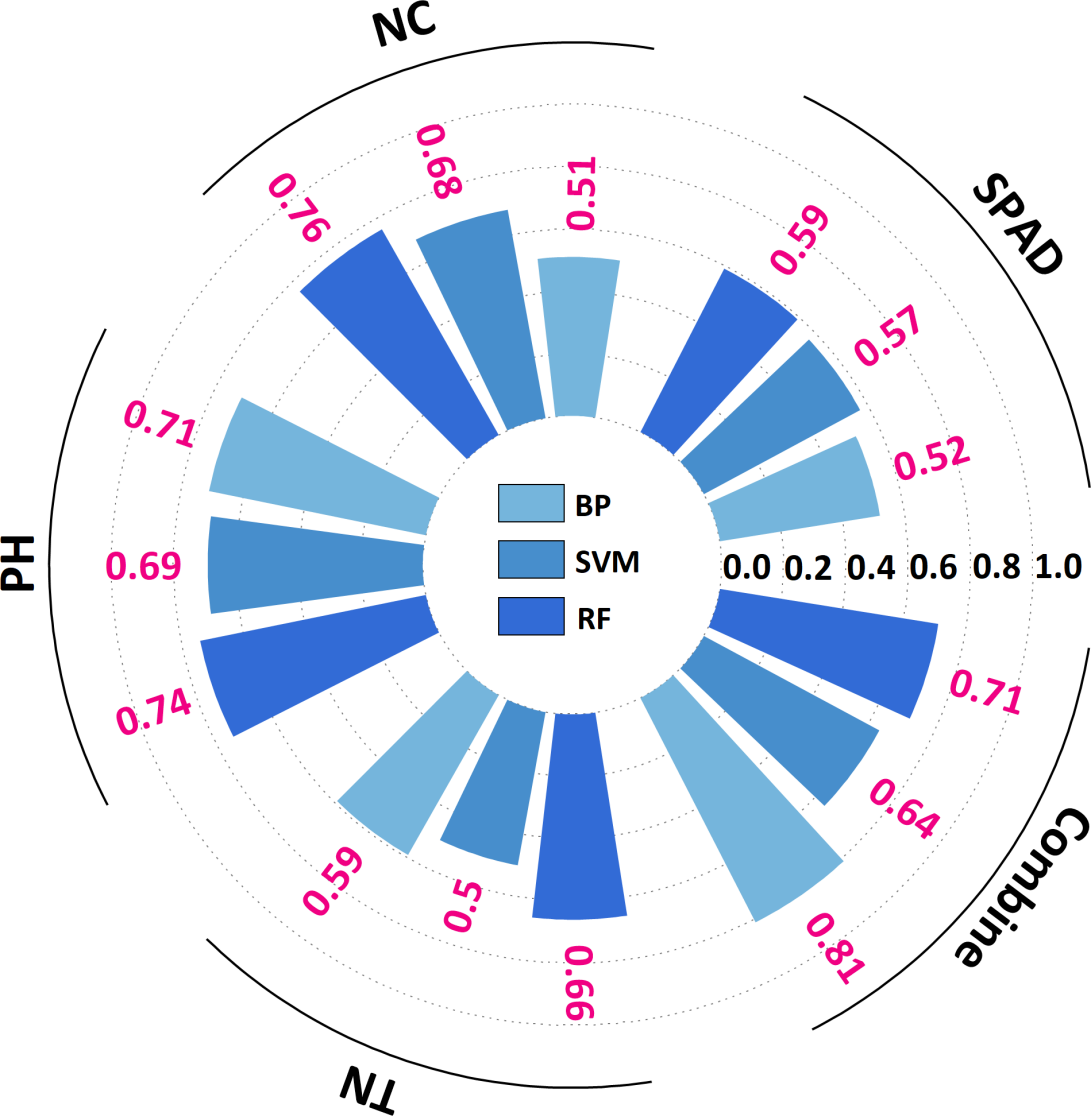
Radial bar chart of *G. uralensis* yield prediction based on phenotypic indicators. PH stand for plant height; TN stand for tiller number; SPAD stand for soil and plant analysis development value; NC stand for nitrogen content. BP stand for back propagation neural network; SVM stand for support vector machine; RF stand for random forest.

The yield prediction study (**Table 6**) revealed a substantial correlation (*R²* > 0.40) between the prediction models of SPAD, NC, TN, and PH and actual yield. The prediction accuracy of multi-indicator models surpassed that of single-indicator models. The BP algorithm exhibited the highest level of accuracy (*R²* = 0.81, *RMSE* = 13.78). The RF algorithm demonstrated the highest performance in all single-indicator predictions. The prediction accuracy of PH and NC was the highest (*R²* = 0.74, *RMSE* = 9.29; *R²* = 0.76, *RMSE* = 8.67), whereas TN and SPAD exhibited slightly lower prediction accuracy (*R²* = 0.59, *RMSE* = 11.10; *R²* = 0.66, *RMSE* = 10.35; **Figure 7**). Bar charts and scatter plots of the prediction results are given in **Supplementary Figures S4** and **S5**. Among the four PIs, PH demonstrated the optimal comprehensive predictive capacity. Concurrently, NC, TN, and SPAD functioned as auxiliary modeling instruments.

**Table 6:** Phenotypic indicators predict yield results.

Phenotypic indicators	Algorithm	R^2^	RMSE	MAE	MBE	Equation
SPAD	BP	0.52	8.49	9.41	-1.76	y=0.49x+24.96
	SVM	0.57	12.11	7.51	-0.03	y=0.57x+27.47
	RF	0.59	11.10	8.51	0.05	y=0.46x+34.81
NC/mg*g^-1^	BP	0.51	11.02	8.80	-1.33	y=0.46x+32.49
	SVM	0.68	10.11	9.19	-3.58	y=0.48x+29.51
	RF	0.76	8.67	8.25	0.35	y=0.46x+37.11
PH/cm	BP	0.71	12.11	8.66	1.78	y=0.75x+18.45
	SVM	0.69	12.17	7.25	0.58	y=0.75x+17.99
	RF	0.74	9.29	8.45	0.42	y=0.62x+24.06
TN	BP	0.59	14.97	8.98	0.38	y=0.72x+19.98
	SVM	0.50	10.14	17.60	-0.07	y=0.10x+60.53
	RF	0.66	10.35	9.33	0.56	y=0.57x+29.80
Combine	BP	0.81	13.78	5.98	-0.04	y=0.57x+28.77
	SVM	0.64	2.66	5.60	0.03	y=0.18x+53.68
	RF	0.71	7.91	6.95	0.07	y=0.57x+29.80

PH stand for plant height; TN stand for tiller number; SPAD stand for soil and plant analysis development value; NC stand for nitrogen content. BP stand for back propagation neural network; SVM stand for support vector machine; RF stand for random forest.

## 4 Discussion

### 4.1 Effect of water and nitrogen treatments on *G. uralensis* growth​

Water and nitrogen are essential substrates for plant physiological metabolism, synergistically regulating chlorophyll synthesis, morphological development, and yield formation. Our study demonstrated that water–nitrogen deficit significantly decreased PH, TN, SPAD, and NC in *G. uralensis* compared with the CK, leading to dual losses in yield and quality. This is consistent with crop stress response patterns reported by Tan and Liu (Tan et al., 2023; Liu et al., 2020), mechanistically driven by multiphysiological interactions. In morphological development, water stress inhibits root elongation and lateral root differentiation by suppressing cell turgor and auxin synthesis (Condorelli et al., 2018; Vurro et al., 2023). Nitrogen deficiency restricts protein synthesis, curbing leaf expansion and tillering (Yang et al., 2017), a pattern validated across herbaceous plants. Photosynthetically, water stress reduces stomatal conductance, limiting CO_2_ assimilation efficiency (Bao et al., 2025; Kaya and Ergin, 2025), whereas nitrogen deficiency diminishes light-harvesting capacity by lowering chloroplast density and abundance of PSII reaction center (Chen et al., 2024b), with similar responses observed in cereal crops. During yield-quality formation, water deficiency inhibits root expansion and secondary metabolite synthesis (Wang et al., 2024a), whereas nitrogen deficiency decreases photosynthetic pigment content and critical enzyme activity (Maluleke and Thobejane, 2025), collectively compromising resource allocation. These physiological shifts manifest as spectral signatures at the canopy scale, consistent with mechanisms identified by Jin and Ding (Jin et al., 2023; Ding et al., 2025) reporting that spectral changes directly reflect alterations in plant pigments and structure. UAV-based multispectral technology captures these dynamics in real time, leveraging strong correlations between VIs and PIs to quantify cumulative water–nitrogen stress effects. This enables synchronous diagnosis of PH, TN, chlorophyll, and nitrogen dynamics, supporting precise water–nutrient regulation (Li et al., 2024a, 2024).

### 4.2 Model evaluation​

Our study developed efficient, nondestructive growth monitoring and yield prediction techniques by integrating UAV-derived VIs and PIs. Pearson correlation analysis revealed high redundancy (*r* > 0.99) among NDVI, EVI, and four other VIs due to shared near-infrared–red band foundations and statistical homology (Ma et al., 2023). RF outperformed BP and SVM in capturing nonlinear relationships through multitree integration, with feature importance ranking aligning with agronomic decision needs (Wu et al., 2025). A 7:3 training–testing ratio ensured robust learning (70% train set) and minimized accuracy fluctuations across growth stages (30% test set) (Zhou et al., 2024; He et al., 2025). After comprehensive optimization of VI selection, algorithms, and data partitioning, the constructed model provided precise water–nutrient decision support for *G. uralensis* cultivation, achieving synergistic yield–quality enhancement.

**Figure 8:**
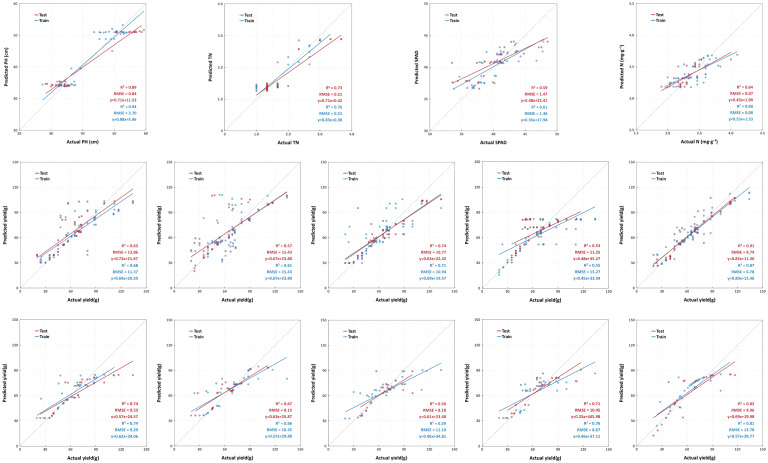
Fitted scatter plot of the model. (**a**, top) Scatter plots of optimal prediction performance for phenotypic indicators based on vegetation indices (left to right): BP - GLI - PH, RF - RDVI - TN, RF - EXG - SPAD, RF - GRRI - NC. (**b**, center): Scatter plots of optimal yield prediction performance based on vegetation indices (left to right): RF - BRVI, SVM - EXG, RF - GRAY, SVM - NDRE, RF - Combine. (**c**, bottom): Scatter plots of optimal yield prediction performance based on phenotypic indicators (left to right): RF - PH, RF - TN, RF - SPAD, RF - NC, BP - Combine. PH stand for plant height; TN stand for tiller number; SPAD stand for soil and plant analysis development value; NC stand for nitrogen content. EXG stand for excess green index; GLI stand for green leaf index; NDRE stand for normalized difference red - edge index; GRAY stand for gray level index; GRRI stand for green - red ratio index; BRVI stand for simple blue ratio index; RDVI stand for renormalized difference vegetation index. BP stand for back propagation neural network; SVM stand for support vector machine; RF stand for random forest.

UAV-based phenotyping technology, characterized by nondestructiveness, efficiency, and high precision, remains pivotal in crop growth monitoring (Sapkota and Paudyal, 2023; Bai et al., 2023). Our integrated models achieved high-precision inversion of PH, TN, SPAD, and NC (0.42 ≤ R² ≤ 0.94), with RF coupled to the EXG demonstrating exceptional stability across all four PIs (**Figure 8a**). Early-stage PH prediction errors originated from vertical projection deviations caused by prostrate stem morphology in seedlings, later mitigated by structural stability (Wang et al., 2024b). TN exhibited strong correlation with VIs due to limited variability, enabling high accuracy through multisample averaging (Lu et al., 2024). SPAD and NC predictions were constrained by canopy structural issues, where spectral mixing errors at current resolutions led to slightly inferior accuracy, yet markedly surpassed traditional sampling methods (Xu et al., 2023). These results validated the superiority of UAV multispectral technology for growth monitoring, providing actionable insights for real-time precise water–nutrient regulation.

Yield prediction is crucial for agricultural management, determining economic returns and resource optimization (Goodwin et al., 2018). Leveraging UAV high-resolution, full-phenology data, BP, SVM, and RF models using VIs and PIs achieved high-precision yield forecasts (VI models: 0.41 ≤ *R²* ≤ 0.87; PI models: 0.50 ≤ *R²* ≤ 0.81), with VIs slightly outperforming PIs (**Figures 8b, c**). BP and RF algorithms proved suitable for this study, and multiparameter joint prediction enhanced accuracy beyond single-parameter approaches, a widespread pattern in crop yield modeling (Shu et al., 2023; McBreen et al., 2025). Though VIs matched PIs in accuracy through multispectral recognition, canopy structural interference particularly occlusion and mixed-pixel effects persisted as constraints (Sun et al., 2024; Hui et al., 2024). Innovative integration of photosynthetic parameters (SPAD and NC) with morphological traits (PH and TN) significantly improved model accuracy (*R²* ≥ 0.64). This multi-indicator approach confirmed PH as the important predictor while revealing the auxiliary monitoring value of photosynthetic parameters (Ji et al., 2022; Dai et al., 2024). Extending multi-phenotype framework to medicinal plants (Singh et al., 2019), this study enabled UAV-guided variable irrigation and nitrogen topdressing, advancing cultivation from “empirical management” to “demand-oriented supply” for real-time water–nutrient decisions.

### 4.3 Model applicability and limitations​

This study constructed a full-growth-stage monitoring and yield prediction model for *G. uralensis* by fusing UAV multispectral technology with VIs and PIs. The RF algorithm combined with the EXG demonstrated high versatility in growth monitoring and yield prediction modeling. By integrating photosynthetic parameters and PIs, this system accurately deciphered real-time field variations to formulate dynamic water–nutrient regulation schemes, thereby achieving resource conservation and quality enhancement in sandy loam soils of Inner Mongolia. Technical advantages included early monitoring capability through EXG-based VIs, reduced errors via UAV precision technology, and water–nutrient management strategies covering critical reproductive cycles. Compared with traditional methods, this system significantly improves monitoring efficiency, minimizes sampling-induced tissue damage, and proves better suited for large-scale agricultural production.

We validated the field applicability of models. However, this study only examined nitrogen fertilization; the impacts of phosphorus and potassium on growth and yield require further investigation. The water–nitrogen gradient was designed with a CK-based progressive decrease to simulate arid wild conditions in Inner Mongolia, aiming to provide data support for water and fertilizer conservation cultivation. Future studies should explore the effects of excessive irrigation and excessive fertilization. We successfully established multiple high-precision models at a 50-m flight height for real-time monitoring of water–nutrient demand dynamics. However, developing universally applicable growth monitoring models remains challenging because of the uncontrolled nature of soil hydrochemical properties and the trade-off between efficiency and accuracy at this altitude. Hyperspectral or higher-resolution sensors may help in alleviating these constraints. Future studies should investigate the impact of UAV flight altitude on monitoring precision and efficiency.

## 5 Conclusions

This study developed an advanced multispectral growth monitoring system for *G. uralensis* using UAVs, achieving *R²* = 0.94. The study encompassed the construction of a water–fertilizer deficiency model and its subsequent field application, thus demonstrating the comprehensive nature of the system. Furthermore, the construction of stable yield prediction models based on VIs and PIs was successfully achieved (*R²* = 0.87; 0.81). The results of this study suggested that the RF algorithm exhibited greater generalizability in model construction for both growth monitoring and yield prediction, whereas the EXG demonstrated greater applicability for growth monitoring. The proposed framework provided a valuable reference for the application of UAV remote sensing in smart agriculture, assisting *G. uralensis* cultivators and botanists in optimizing management decisions. With the rapid development of UAV remote sensing technology, UAVs equipped with multisensor systems would play an important role in the sustainable exploitation and utilization of medicinal plant resources.

## Data Availability

The original contributions presented in the study are included in the article/**Supplementary Material**. Further inquiries can be directed to the corresponding authors.

## References

[B1] Ali H. Gao W. Zeng W. Li S. Zhang L. Wang W.. (2024). Remote sensing estimation of sugar beet SPAD based on un-manned aerial vehicle multispectral imagery. PloS One 19, e03000565. doi: 10.1371/journal.pone.0300056, PMID: 38905187 PMC11192409

[B2] Andvaag E. Krys K. Shirrtliffe S. J. Stavness I. (2024). Counting canola: toward generalizable aerial plant detection models. Plant Phenomics 6, 268. doi: 10.34133/plantphenomics.0268, PMID: 39525981 PMC11543947

[B3] Ashrafuzzaman M. Wang X. Shen Y. Tian P. Wu M. Li Z.. (2025). OBM-RFEcv: An adaptive ensemble model for monitoring key growth indicators of Gerbera using multi-spectral image fusion features. PloS One 20, e0322851. doi: 10.1371/journal.pone.0322851, PMID: 40392889 PMC12091738

[B4] Bai X. Fang H. He Y. Zhang J. Tao M. Wu Q.. (2023). Dynamic UAV phenotyping for rice disease resistance analysis based on multisource data. Plant Phenomics 5, 19. doi: 10.34133/plantphenomics.0019, PMID: 37040287 PMC10076055

[B5] Bao Q. Wu Y. Du H. Wang Y. Zhang Y. (2025). Phenotypic physiological and metabolomic analyses reveal crucial metabolic pathways in quinoa (Chenopodium quinoa willd.) in response to PEG-6000 induced drought stress. Int. J. Mol. Sci. 26, 2599. doi: 10.3390/ijms26062599, PMID: 40141239 PMC11942229

[B6] Basso M. Stocchero D. Ventura Bayan Henriques R. Vian A. L. Bredemeier C. Konzen A. A.. (2019). Proposal for an embedded system architecture using a GNDVI algorithm to support UAV-based agrochemical spraying. Sensors 19, 5397. doi: 10.3390/s19245397, PMID: 31817832 PMC6960772

[B7] Chen J. LI Q. Jiang D. (2024a). From images to loci: applying 3D deep learning to enable multivariate and multitemporal digital phenotyping and mapping the genetics underlying nitrogen use efficiency in wheat. Plant Phenomics 6. doi: 10.34133/plantphenomics.0270, PMID: 39703939 PMC11658601

[B8] Chen L.-H. XU M. Cheng Z. Yang L.-T. (2024b). Effects of nitrogen deficiency on the photosynthesis, chlorophyll a fluorescence, antioxidant system, and sulfur compounds in oryza sativa. Int. J. Mol. Sci. 25, 10409. doi: 10.3390/ijms251910409, PMID: 39408737 PMC11476759

[B9] Condorelli G. E. Maccaferri M. Newcomb M. Andrade-Sanchenz P. White J. W. French A. N.. (2018). Comparative aerial and ground based high throughput phenotyping for the genetic dissection of NDVI as a proxy for drought adaptive traits in durum wheat. Front. Plant Sci. 9. doi: 10.3389/fpls.2018.00893, PMID: 29997645 PMC6028805

[B10] Dai Y. Yu S. E. Ma T. Ding J. Chen K. Zeng G.. (2024). Improving the estimation of rice above-ground biomass based on spatio-temporal UAV imagery and phenological stages. Front. Plant Sci. 15. doi: 10.3389/fpls.2024.1328834, PMID: 38774220 PMC11106403

[B11] Deveerasetty K. K. Abbas N. Liu X. Iqbal J. (2024). A flexible mixed-optimization with H∞ control for coupled twin rotor MIMO system based on the method of inequality (MOI)- An experimental study. PloS One 19, e0300305. doi: 10.1371/journal.pone.0300305, PMID: 38517873 PMC10959396

[B12] Ding G. Shi Y. Xie K. Li H. Xiao G. (2025). Genome-wide identification and expression analysis of bHLH gene family revealed their potential roles in abiotic stress response, anthocyanin biosynthesis and trichome formation in *Glycyrrhiza uralensis* . Front. Plant Sci. 15. doi: 10.3389/fpls.2024.1485757, PMID: 39906234 PMC11790457

[B13] Du P. Yin B. Zhou S. Li Z. Zhang X. Cao Y.. (2022). Melatonin and dopamine mediate the regulation of nitrogen uptake and metabolism at low ammonium levels in Malus hupehensis. Plant Physiol. Biochem. 171. doi: 10.3389/fpls.2024.1485757, PMID: 35007949

[B14] Duque A. F. Patino D. Colorado J. D. Petro E. Rebolledo M. C. Mondragon I. F.. (2023). Characterization of rice yield based on biomass and SPAD-based leaf nitrogen for large genotype plots. Sensors 23, 5917. doi: 10.3390/s23135917, PMID: 37447767 PMC10347115

[B15] Filonchyk M. Xie D. Zeng F. Liu B. Fang Q. Dong Y.. (2025). Evaluating land-cover change and land subsidence in coal fire zones: Insights from multi-source monitoring. PloS One 20, e0322284. doi: 10.1371/journal.pone.0322284, PMID: 40435205 PMC12118845

[B16] Goodwin A. W. Lindsey L. E. Harrison S. K. Paul P. A. (2018). Estimating wheat yield with normalized difference vegetation index and fractional green canopy cover. Crop Forage Turfgrass Manage. 4, 1–6. doi: 10.2134/cftm2018.04.0026

[B17] He Q. Zhan J. Liu X. Dong C. Tian D. Fu Q. (2025). Multispectral polarimetric bidirectional reflectance research of plant canopy. Opt. Laser Eng. 184, 108688. doi: 10.1016/j.optlaseng.2024.108688

[B18] Hu X. Du Z. Wang F. (2024). Research on detection method of photovoltaic cell surface dirt based on image processing technology. Sci. Rep. 14, 16842. doi: 10.1038/s41598-024-68052-z, PMID: 39039184 PMC11263714

[B19] Huang C. Liu K. Ma T. Xue H. Wang P. Li L.. (2025). Analysis of the impact mechanisms and driving factors of urban spatial morphology on urban heat islands. Sci. Rep. 15, 18589. doi: 10.1038/s41598-025-04025-0, PMID: 40425735 PMC12116756

[B20] Hui D. Jang C. Namoi N. Wolske E. Wasonga D. Behnke G.. (2024). Integrating plant morphological traits with remote-sensed multispectral imageries for accurate corn grain yield prediction. PloS One 19, e0297027. doi: 10.1371/journal.pone.0297027, PMID: 38564609 PMC10986971

[B21] Itoh A. Njane S. N. Hirafuji M. Guo W. (2024). PREPs: an open-source software for high-throughput field plant phenotyping. Plant Phenomics 6, 221. doi: 10.34133/plantphenomics.0221, PMID: 39130162 PMC11310773

[B22] Ji Y. Chen Z. Cheng Q. Liu R. Li M. Yan X.. (2022). Estimation of plant height and yield based on UAV imagery in faba bean (Vicia faba L.). Plant Methods 18, 26. doi: 10.1186/s13007-022-00861-7, PMID: 35246179 PMC8897926

[B23] Jiang M. Zhao S. Yang S. Lin X. He X. Wei X.. (2020). An “essential herbal medicine”—licorice: A review of phytochemicals and its effects in combination preparations. J. Ethnopharmacol. 249, 112439. doi: 10.1016/j.jep.2019.112439, PMID: 31811935

[B24] Jin E. J. Yoon J.-H. Lee H. Bae E. J. Yong S. H. Choi M. S. (2023). Evaluation of drought stress level in Sargent’s cherry (Prunus sargentii Rehder) using photosynthesis and chlorophyll fluorescence parameters and proline content analysis. Peer J. 11, e15954. doi: 10.7717/peerj.15954, PMID: 37842053 PMC10576498

[B25] Johansen K. Morton M. J. L. Malbeteau Y. Aragon B. Al-Mashharawi S. Ziliani M. G.. (2020). Predicting biomass and yield in a tomato phenotyping experiment using UAV imagery and random forest. Front. Artif. Intell. 3. doi: 10.3389/frai.2020.00028, PMID: 33733147 PMC7861253

[B26] Jurevičius L. Punys P. Šadzevičius R. Kasiulis E. (2022). Monitoring dewatering fish spawning sites in the reservoir of a large hydropower plant in a lowland country using unmanned aerial vehicles. Sensors 23, 303. doi: 10.3390/s23010303, PMID: 36616901 PMC9824071

[B27] Kaya G. Ergin N. (2025). Classification of red beet and sugar beet for drought tolerance using morpho-physiological and stomatal traits. Peer J. 13, e19133. doi: 10.7717/peerj.19133, PMID: 40130173 PMC11932113

[B28] Lee K. Sudduth K. A. Zhou J. (2024). Evaluating UAV-based remote sensing for hay yield estimation. Sensors 24, 5326. doi: 10.3390/s24165326, PMID: 39205020 PMC11360442

[B29] Li J. Cao X. Jia X. Liu L. Cao H. Qin W.. (2021). Iron deficiency leads to chlorosis through impacting chlorophyll synthesis and nitrogen metabolism in areca catechu L. Front. Plant Sci. 12. doi: 10.3389/fpls.2021.710093, PMID: 34408765 PMC8365612

[B30] Li H. Li Q. Yu C. Luo S. (2025a). Unified estimation of rice canopy leaf area index over multiple periods based on UAV multispectral imagery and deep learning. Plant Methods 21, 73. doi: 10.1186/s13007-025-01398-1, PMID: 40442795 PMC12123809

[B31] Li W. Pan K. Liu W. Xiao W. Ni S. Shi P.. (2024b). Monitoring maize canopy chlorophyll content throughout the growth stages based on UAV MS and RGB feature fusion. Agriculture 14, 1265. doi: 10.3390/agriculture14081265

[B32] Li J. Wu W. Zhao C. Bai X. Dong L. Tan Y.. (2025b). Effects of solar elevation angle on the visible light vegetation index of a cotton field when extracted from the UAV. Sci. Rep. 15, 18497. doi: 10.1038/s41598-025-00992-6, PMID: 40425651 PMC12117064

[B33] Li D. Yang S. Du Z. Xu X. Zhang P. Yu K.. (2024a). Application of unmanned aerial vehicle optical remote sensing in crop nitrogen diagnosis: A systematic literature review. Comput. Electron. Agric. 227, 109565. doi: 10.1016/j.compag.2024.109565

[B34] Liao M. Wang Y. Chu N. Li S. Zhang Y. Lin D. (2025). Mature rice biomass estimation using UAV-derived RGB vegetation indices and growth parameters. Sensors 25, 2798. doi: 10.3390/s25092798, PMID: 40363244 PMC12074424

[B35] Liu X. Yin C. Xiang L. Jiang W. Xu S. Mao Z. (2020). Transcription strategies related to photosynthesis and nitrogen metabolism of wheat in response to nitrogen deficiency. BMC Plant Biol. 20, 448. doi: 10.1186/s12870-020-02662-3, PMID: 33003994 PMC7528333

[B36] Lu X. Shen Y. Xie J. Yang X. Shu Q. Chen S.. (2024). Phenotyping of panicle number and shape in rice breeding materials based on unmanned aerial vehicle imagery. Plant Phenomics 6, 265. doi: 10.34133/plantphenomics.0265, PMID: 39449974 PMC11499587

[B37] Lu D. Yang Y. Du Y. Zhang L. Yang Y. Tibenda J. J.. (2023). The potential of glycyrrhiza from “Medicine food homology” in the fight againstDigestive system tumors. Molecules 28, 7719. doi: 10.3390/molecules28237719, PMID: 38067451 PMC10708138

[B38] Ma X. Ding J. Wang T. Lu L. Sun H. Zhang F.. (2023). A pixel dichotomy coupled linear kernel-driven model for estimating fractional vegetation cover in arid areas from high-spatial-resolution images. IEEE Trans. Geosci. Remote Sens. 61, 1–15. doi: 10.1109/TGRS.2023.3289093

[B39] Maluleke M. K. Thobejane K. R. (2025). Physiology, yield and nutritional contribution of hemp (Cannabis sativa L.) grown under different fertiliser types and environments. J. Cannabis Res. 7, 17. doi: 10.1186/s42238-025-00273-z, PMID: 40121494 PMC11929295

[B40] Mcbreen J. Babar M. A. Jarquin D. Ampatzidis Y. Khan N. Kunwar S.. (2025). Enhancing genomic-based forward prediction accuracy in wheat by integrating UAV-derived hyperspectral and environmental data with machine learning under heat-stressed environments. Plant Genome 18, e20554. doi: 10.1002/tpg2.20554, PMID: 39779660 PMC11711122

[B41] Okada M. Barras C. Toda Y. Hamazaik K. Ohmori Y. Yamasaki Y.. (2024). High-throughput phenotyping of soybean biomass: conventional trait estimation and novel latent feature extraction using UAV remote sensing and deep learning models. Plant Phenomics 6, 244. doi: 10.34133/plantphenomics.0244, PMID: 39252878 PMC11382017

[B42] Plett D. C. Ranathunge K. Melino V. J. Kuya N. Uga Y. Kronzucker H. J.. (2020). The intersection of nitrogen nutrition and water use in plants: new paths toward improved crop productivity. J. Exp. Bot. 71, 4452–4468. doi: 10.1093/jxb/eraa049, PMID: 32026944 PMC7382376

[B43] Pun Magar L. Sandifer J. Khatri D. Poudel S. Kc S. Gyawali B.. (2025). Plant height measurement using UAV-based aerial RGB and LiDAR images in soybean. Front. Plant Sci. 16. doi: 10.3389/fpls.2025.1488760, PMID: 39949411 PMC11821976

[B44] Roth L. Binder M. Kirchgessner N. Tschurr F. Yates S. Hund A.. (2024). From neglecting to including cultivar-specific per se temperature responses: extending the concept of thermal time in field crops. Plant Phenomics 6, 185. doi: 10.34133/plantphenomics.0185, PMID: 38827955 PMC11142864

[B45] Sapkota S. Paudyal D. R. (2023). Growth monitoring and yield estimation of maize plant using unmanned aerial vehicle (UAV) in a hilly region. Sensors 23, 5432. doi: 10.3390/s23125432, PMID: 37420599 PMC10300992

[B46] Shahzad A. N. Rizwan M. Asghar M. G. Qureshi M. K. Bukhari S. A. H. Kiran A.. (2019). Early maturing Bt cotton requires more potassium fertilizer under water deficiency to augment seed-cotton yield but not lint quality. Sci. Rep. 9, 7378. doi: 10.1038/s41598-019-43563-2, PMID: 31089147 PMC6517391

[B47] Sharma V. Honkavaara E. Hayden M. Kant S. (2024). UAV remote sensing phenotyping of wheat collection for response to water stress and yield prediction using machine learning. Plant Stress 12, 100464. doi: 10.1016/j.stress.2024.100464

[B48] Shen L. Ding G. Jackson R. Ali M. Liu S. Mitchell A.. (2024). GSP-AI: an AI-powered platform for identifying key growth stages and the vegetative-to-reproductive transition in wheat using trilateral drone imagery and meteorological data. Plant Phenomics 6, 255. doi: 10.34133/plantphenomics.0255, PMID: 39386010 PMC11462051

[B49] Shi H. Liu Z. Li S. Jin M. Tang Z. Sun T.. (2024). Monitoring soybean soil moisture content based on UAV multispectral and thermal-infrared remote-sensing information fusion. Plants 13, 2417. doi: 10.3390/plants13172417, PMID: 39273901 PMC11396815

[B50] Shu M. Li Q. Ghafoor A. Zhu J. Li B. Ma Y.. (2023). Using the plant height and canopy coverage to estimation maize aboveground biomass with UAV digital images. Eur. J. Agron. 151, 126957. doi: 10.1016/j.eja.2023.126957

[B51] Singh D. Wang X. Kumar U. Gao L. Noor M. Imtiaz M.. (2019). High-throughput phenotyping enabled genetic dissection of crop lodging in wheat. Front. Plant Sci. 10. doi: 10.3389/fpls.2019.00394, PMID: 31019521 PMC6459080

[B52] Song Z. Tomasetto F. Niu X. Yan W. Q. Jiang J. Li Y.. (2022). Enabling breeding selection for biomass in slash pine using UAV-based imaging. Plant Phenomics, 9783785. doi: 10.34133/2022/9783785, PMID: 35541565 PMC9057123

[B53] Sumnall M. J. Carter D. R. Albaugh T. J. Cook R. L. Campoe O. C. Rubilar R. A.. (2024). Evaluating the influence of row orientation and crown morphology on growth of pinus taeda L. with drone-based airborne laser scanning. Plant Phenomics 6, 264. doi: 10.34133/plantphenomics.0264, PMID: 39444660 PMC11496608

[B54] Sun C. Zhang W. Zhao G. Wu Q. Liang W. Ren N.. (2024). Mapping rapeseed (Brassica napus L.) aboveground biomass in different periods using optical and phenotypic metrics derived from UAV hyperspectral and RGB imagery. Front. Plant Sci. 15. doi: 10.3389/fpls.2024.1504119, PMID: 39711583 PMC11659009

[B55] Tan W. Li W. Li J. Liu D. Xing W. (2023). Drought resistance evaluation of sugar beet germplasms by response of phenotypic indicators. Plant Signal. Behav. 18, e2192570. doi: 10.1080/15592324.2023.2192570, PMID: 36966541 PMC10044154

[B56] Tang Z. Lu J. Abdelghany A. E. Su P. Jin M. Li S.. (2025). Winter oilseed rape LAI inversion via multi-source UAV fusion: A three-dimensional texture and machine learning approach. Plants 14, 1245. doi: 10.3390/plants14081245, PMID: 40284132 PMC12030242

[B57] Traba J. Gómez-Catasús J. Barrero A. Bustillo-De La Rosa D. Zurdo J. Hervás I.. (2022). Comparative assessment of satellite- and drone-based vegetation indices to predict arthropod biomass in shrub-steppes. Ecol. Appl. 32, e2707. doi: 10.1002/eap.2707, PMID: 35808937 PMC10078389

[B58] Volpato L. Wright E. M. Gomez F. E. (2024). Drone-based digital phenotyping to evaluating relative maturity, stand count, and plant height in dry beans (Phaseolus vulgaris L.). Plant Phenomics 6, 278. doi: 10.34133/plantphenomics.0278, PMID: 39610705 PMC11602537

[B59] Vurro F. Croci M. Impollonia G. Marchetti E. Gracia-Romero A. Bettelli M.. (2023). Field plant monitoring from macro to micro scale: feasibility and validation of combined field monitoring approaches from remote to *in vivo* to cope with drought stress in tomato. Plants 12, 3851. doi: 10.3390/plants12223851, PMID: 38005747 PMC10674827

[B60] Wan L. Zhang J. Dong X. Du X. Zhu J. Sun D.. (2021). Unmanned aerial vehicle-based field phenotyping of crop biomass using growth traits retrieved from PROSAIL model. Comput. Electron. Agric. 187, 106304. doi: 10.1016/j.compag.2021.106304

[B61] Wang Z. Ma Y. Chen P. Yang Y. Fu H. Yang F.. (2022). Estimation of rice aboveground biomass by combining canopy spectral reflectance and unmanned aerial vehicle-based red green blue imagery data. Front. Plant Sci. 13. doi: 10.3389/fpls.2022.903643, PMID: 35712565 PMC9197132

[B62] Wang H. Singh K. D. Poudel H. P. Natarajan M. Ravichandran P. Eisenreich B.. (2024b). Forage height and above-ground biomass estimation by comparing UAV-based multispectral and RGB imagery. Sensors 24, 5794. doi: 10.3390/s24175794, PMID: 39275705 PMC11397825

[B63] Wang B. Yang C. Zhang J. You Y. Wang H. Yang W.. (2024a). IHUP: an integrated high-throughput universal phenotyping software platform to accelerate unmanned-aerial-vehicle-based field plant phenotypic data extraction and analysis. Plant Phenomics 6, 164. doi: 10.34133/plantphenomics.0164, PMID: 39165669 PMC11335093

[B64] Wu T. A. Liu K. Cheng M. Gu Z. Guo W. Jiao X. (2025). Paddy field scale evapotranspiration estimation based on two-source energy balance model with energy flux constraints and UAV multimodal data. Remote Sens. 17, 1662. doi: 10.1016/j.optlaseng.2024.108688

[B65] Xu X. Liu L. Han P. Gong X. Zhang Q. (2022). Accuracy of vegetation indices in assessing different grades of grassland desertification from UAV. Int. J. Environ. Res. Public Health 19, 16793. doi: 10.3390/ijerph192416793, PMID: 36554681 PMC9779174

[B66] Xu S. Xu X. Blacker C. Gaulton R. Zhu Q. Yang M.. (2023). Estimation of leaf nitrogen content in rice using vegetation indices and feature variable optimization with information fusion of multiple-sensor images from UAV. Remote Sens. 15, 854. doi: 10.3390/rs15030854

[B67] Yan B. Hou J. Li W. Luo L. Ye M. Zhao Z.. (2023). A review on the plant resources of important medicinal licorice. J. Ethnopharmacol. 301, 115823. doi: 10.1016/j.jep.2022.115823, PMID: 36220512

[B68] Yang F. Chu T. Zhang Y. Liu X. Sun G. Chen Z.. (2020). Quality assessment of licorice (Glycyrrhiza glabra L.) from different sources by multiple fingerprint profiles combined with quantitative analysis, antioxidant activity and chemometric methods. Food Chem. 324, 126854. doi: 10.1016/j.foodchem.2020.126854, PMID: 32353655

[B69] Yang G. Liu J. Zhao C. Li Z. Huang Y. Yu H.. (2017). Unmanned aerial vehicle remote sensing for field-based crop phenotyping: current status and perspectives. Front. Plant Sci. 8. doi: 10.1016/j.foodchem.2020.126854, PMID: 28713402 PMC5492853

[B70] Yu R. Cao X. Liu J. Nie R. Zhang C. Yuan M.. (2024). Using UAV-based temporal spectral indices to dissect changes in the stay-green trait in wheat. Plant Phenomics 6, 171. doi: 10.34133/plantphenomics.0171, PMID: 38694449 PMC11062509

[B71] Yu F. Jin Z. Guo S. Guo Z. Zhang H. Xu T.. (2022). Research on weed identification method in rice fields based on UAV remote sensing. Front. Plant Sci. 13. doi: 10.3389/fpls.2022.1037760, PMID: 36438154 PMC9681826

[B72] Yueliang G. Ge G. Li X. Ji C. Wang T. Shen T.. (2025). The aboveground biomass estimation of the grain for green program stands using UAV-liDAR and sentinel-2 data. Sensors 25, 2707. doi: 10.3390/s25092707, PMID: 40363146 PMC12074154

[B73] Zanotta D. C. Dong J. Zhang J. Zhang S. Yu Z. Song Z.. (2025). Vegetation extraction through UAV RGB imagery and efficient feature selection. PloS One. doi: 10.6084/m9.figshare.28374311.v1, PMID: 40344042 PMC12063833

[B74] Zentgraf I. Holz M. Monzón Díaz O. R. Lück M. Kramp K. Pusch V.. (2025). How scale affects N2O emissions in heterogeneous fields of a diversified agricultural landscape. Sci. Rep. 15, 11013. doi: 10.1038/s41598-025-95630-6, PMID: 40164655 PMC11958750

[B75] Zhang P. Niu L. Cia M. Chen H. Sun X. (2024). AAUConvNeXt: enhancing crop lodging segmentation with optimized deep learning architectures. Plant Phenomics 6, 182. doi: 10.34133/plantphenomics.0182, PMID: 39698322 PMC11654911

[B76] Zhou Y. Jiao Y. Sun Y. Gao S. (2019). *In vitro* production and distribution of flavonoids in *Glycyrrhiza uralensis* Fisch. J. Food Sci. Technol. 57, 1553–1564. doi: 10.1007/s13197-019-04191-w, PMID: 32180652 PMC7054497

[B77] Zhou G. Li J. Tian Z. Xu J. Bai Y. (2024). The extended stumpf model for water depth retrieval from satellite multispectral images. IEEE J. Sel. Top. Appl. Earth Obs. Remote Sens. 17, 6779–6790. doi: 10.1109/JSTARS.2024.3368761

[B78] Zhu H. Lin C. Liu G. Wang D. Qin S. Li A.. (2024). Intelligent agriculture: deep learning in UAV-based remote sensing imagery for crop diseases and pests detection. Front. Plant Sci. 15. doi: 10.3389/fpls.2024.1435016, PMID: 39512475 PMC11540708

[B79] Zou M. Liu Y. Fu M. Li C. Zhou Z. Meng H.. (2024). Combining spectral and texture feature of UAV image with plant height to improve LAI estimation of winter wheat at jointing stage. Front. Plant Sci. 14. doi: 10.3389/fpls.2023.1272049, PMID: 38235191 PMC10791996

