# Supplementary material for: Integrated diagnostics and time series sensitivity assessment for growth monitoring of a medicinal plant (Glycyrrhiza uralensis Fisch.) based on unmanned aerial vehicle multispectral sensors
Source: Front Plant Sci. 2025 Aug 19;16:1612898. doi: 10.3389/fpls.2025.1612898 (PMC12401903; doi:10.3389/fpls.2025.1612898)
Supplement: Supplementary file 1 [file DataSheet1.pdf]

TABLE S1: The DJI Phantom 4 Multispectral UAV parameters

Parameter	Value
Takeoff Weight	1487 g
Flight Battery Weight / 5870 mAh	468 g
Flight period	27 min
Operating Ambient Temperature Range	0 - 40℃

TABLE S2: The multispectral sensor parameters

Channel	Wavelength (nm)	Bandwidth (nm)
Blue	450	16
Green	560	16
Red	650	16
Red Edge	730	16
Near - Infrared	840	26
