# Supplementary material for: Integrated diagnostics and time series sensitivity assessment for growth monitoring of a medicinal plant (Glycyrrhiza uralensis Fisch.) based on unmanned aerial vehicle multispectral sensors
Source: Front Plant Sci. 2025 Aug 19;16:1612898. doi: 10.3389/fpls.2025.1612898 (PMC12401903; doi:10.3389/fpls.2025.1612898)
Supplement: Supplementary file 2 [file DataSheet2.pdf]

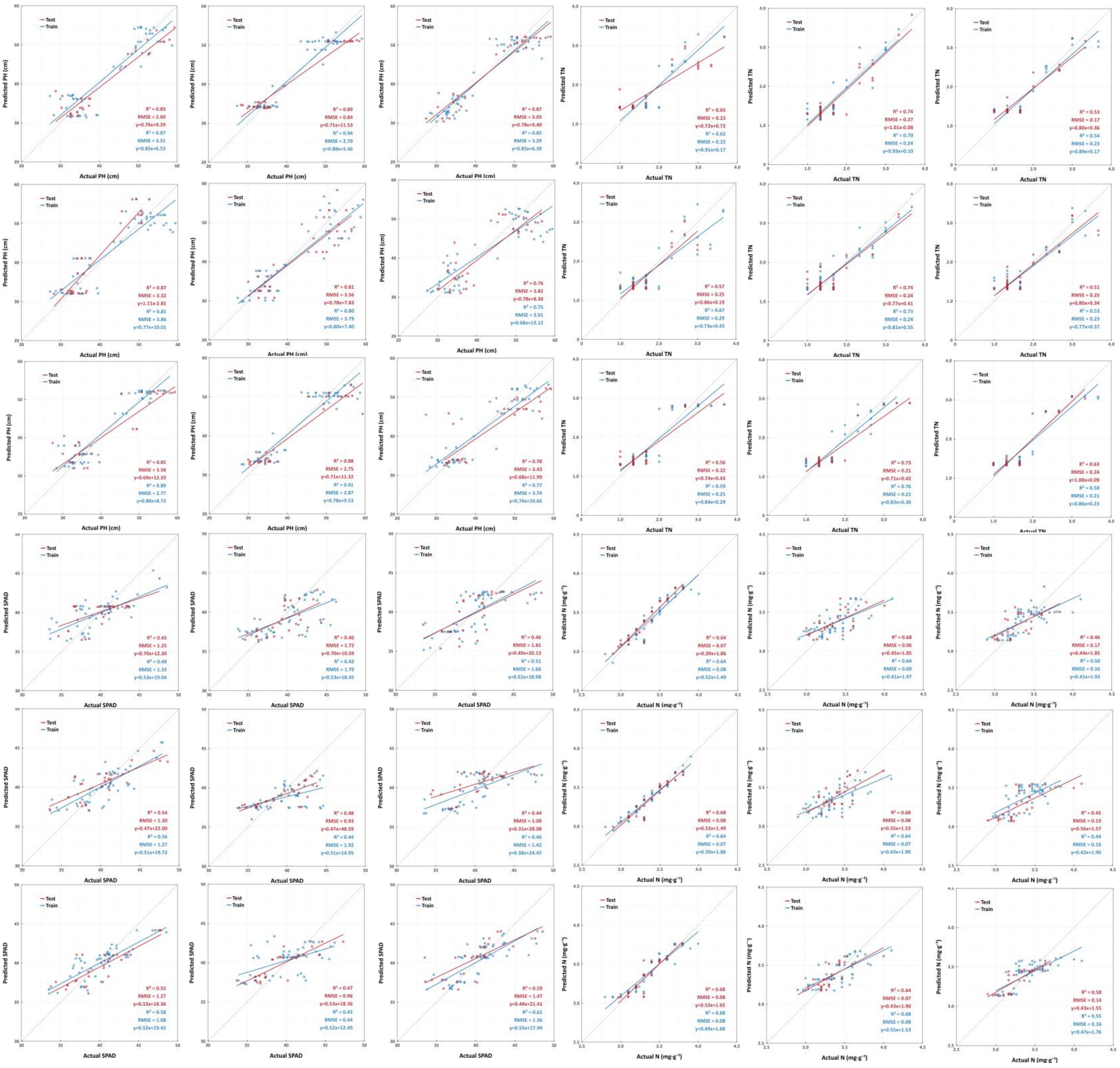

FIGURE S1

Scatter plots of prediction results of *G. uralensis* phenotypic indicators based on vegetation indices. The horizontal arrangement (left to right) represents: plant height (normalized difference red - edge index, green leaf index, excess green index), tiller number (difference vegetation index, renormalized difference vegetation index, excess green index), soil and plant analysis development value (difference vegetation index, renormalized difference vegetation index, excess green index), and nitrogen content (gray level index, green - red ratio index, excess green index); the vertical arrangement (top to bottom) corresponds to algorithms: back propagation neural network, support vector machine and random forest.

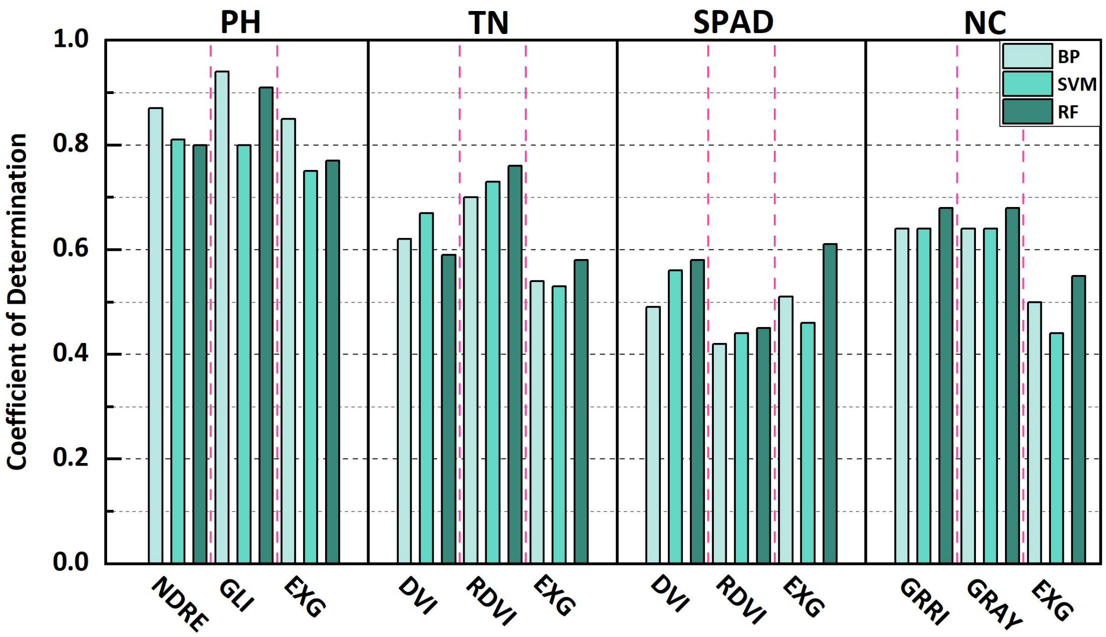

FIGURE S2

Bar plot of prediction results of *G. uralensis* phenotypic indicators based on vegetation indices. PH stand for plant height; TN stand for tiller number; SPAD stand for soil and plant analysis development value; NC stand for nitrogen content. EXG stand for excess green index; GLI stand for green leaf index; NDRE stand for normalized difference red - edge index; GRAY stand for gray level index; GRRI stand for green - red ratio index; RDVI stand for renormalized difference vegetation index; DVI stand for difference vegetation index. BP stand for back propagation neural network; SVM stand for support vector machine; RF stand for random forest.

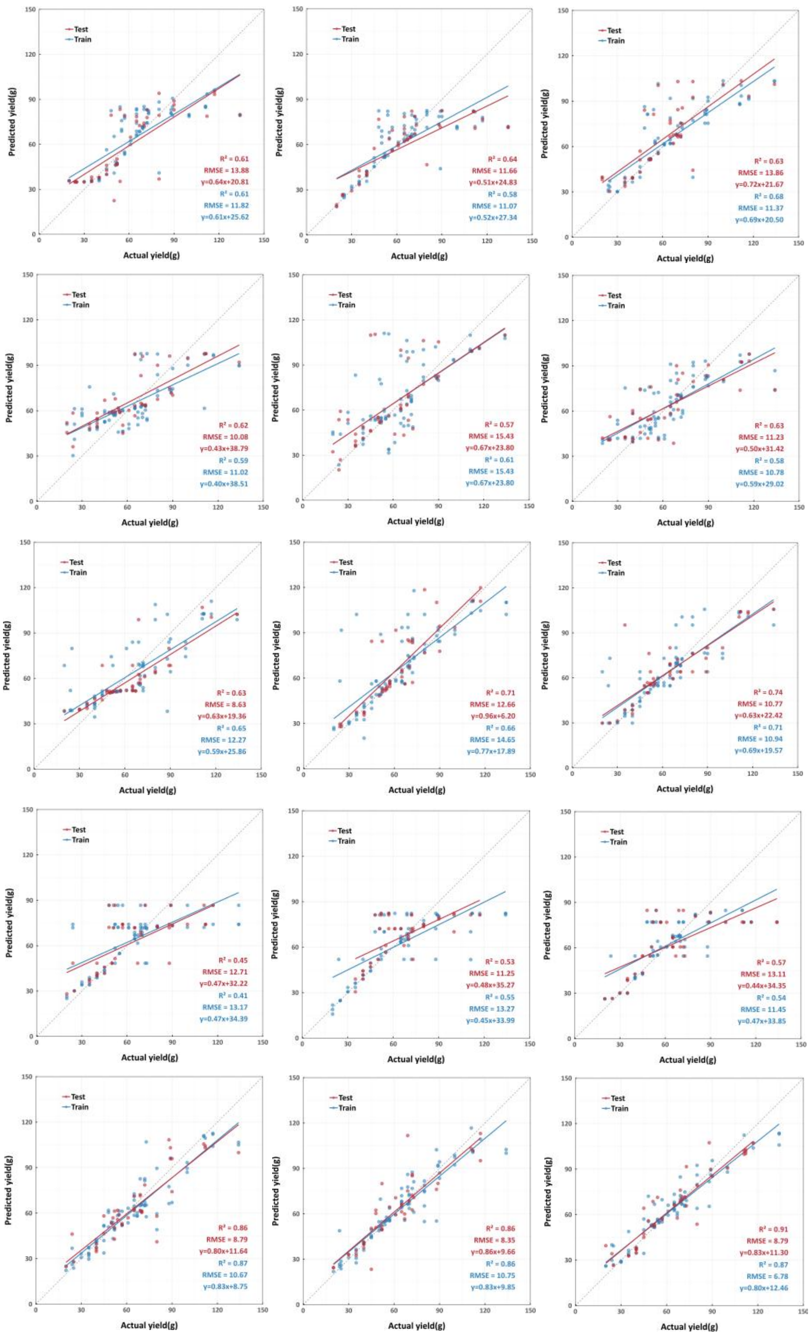

FIGURE S3

Scatter plots of results of *G. uralensis* yield prediction based on vegetation indices. The vertical arrangement (top to bottom) represents: simple blue ratio index, excess green index, gray level index, normalized difference red - edge index, and combine; the horizontal arrangement (left to right) corresponds to algorithms: Back Propagation Neural Network, Support Vector Machine and Random Forest.

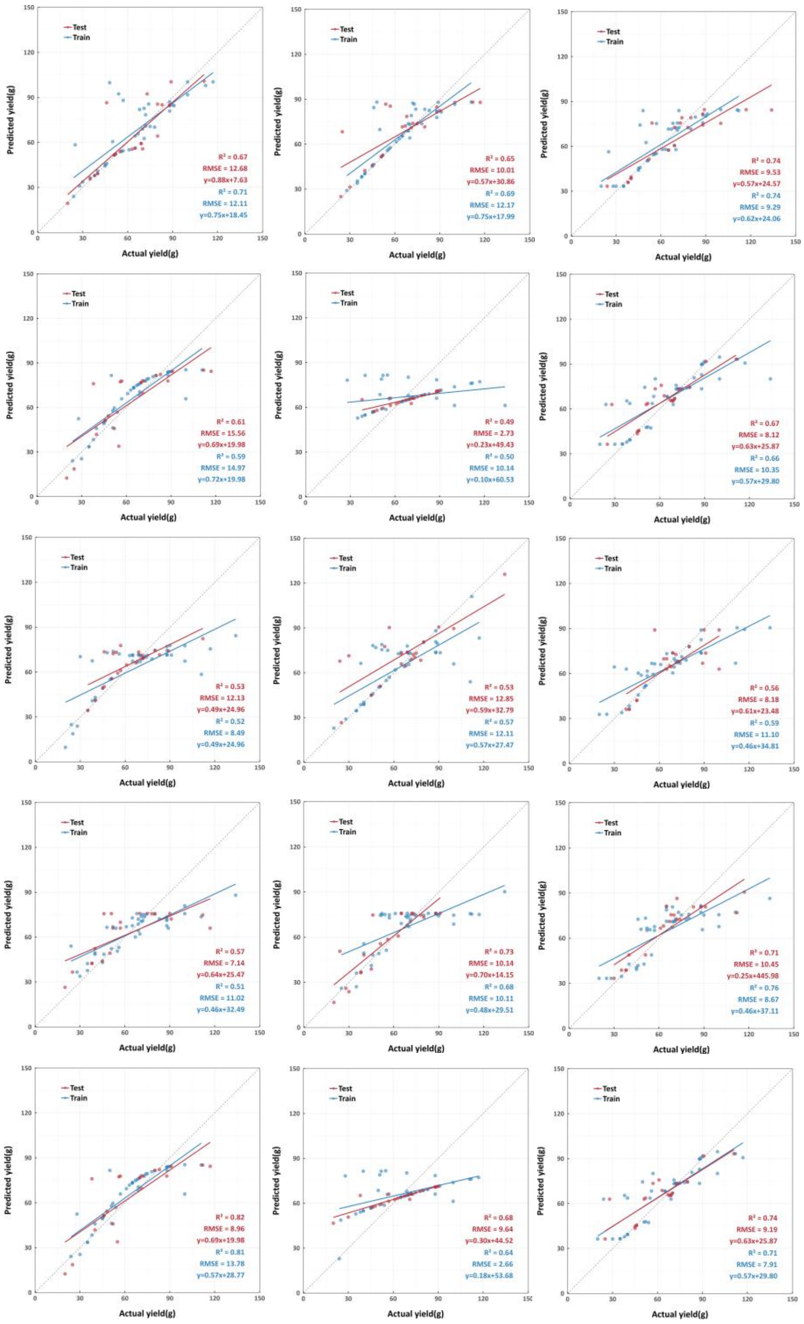

FIGURE S4

Scatter plots of results of *G. uralensis* yield prediction based on phenotypic indicators. The vertical arrangement (top to bottom) represents: plant height, tiller number, soil and plant analysis development value, nitrogen content and combine; the horizontal arrangement (left to right) corresponds to algorithms: back propagation neural network, support vector machine and random forest.

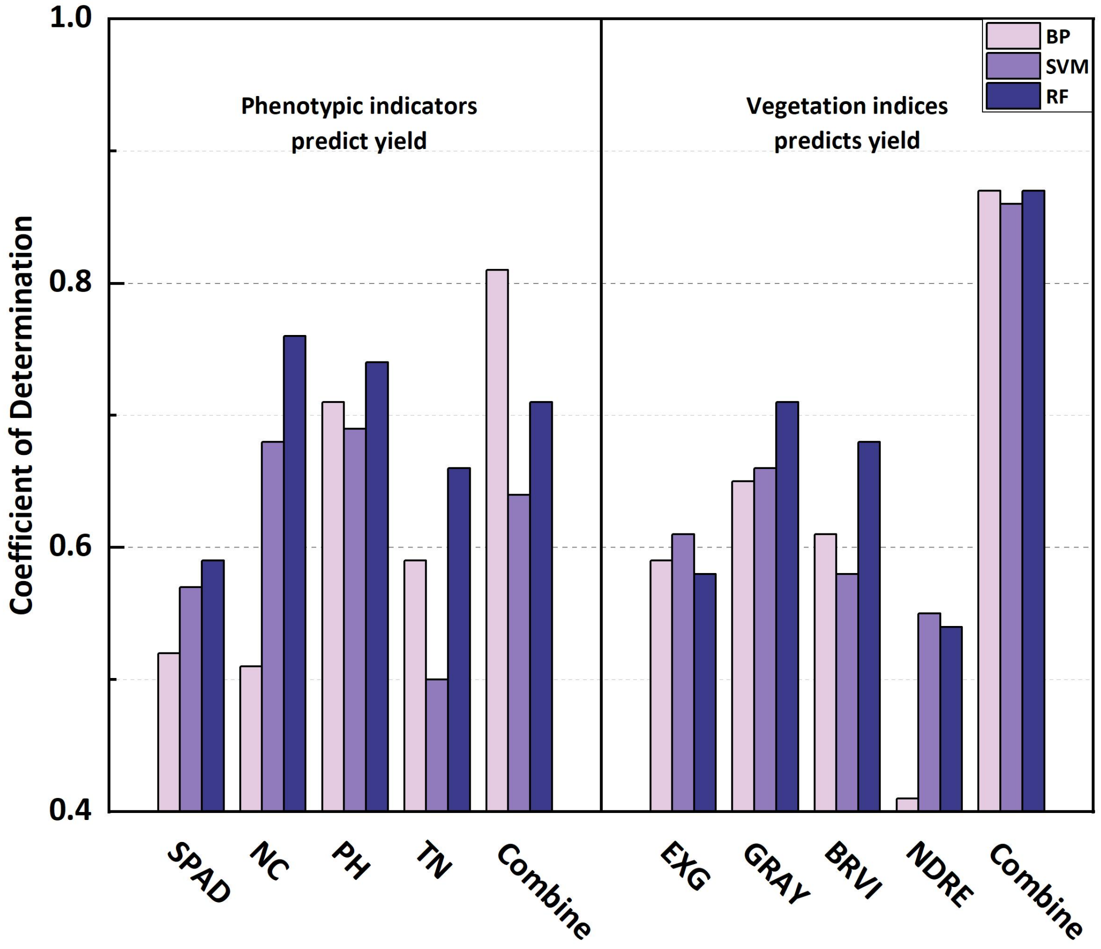

FIGURE S5

Bar plot of results of *G. uralensis* yield prediction. PH stand for plant height; TN stand for tiller number; SPAD stand for soil and plant analysis development value; NC stand for nitrogen content. EXG stand for excess green index; GLI stand for green leaf index; GRAY stand for gray level index; BRVI stand for simple blue ratio index; NDRE stand for normalized difference red - edge index. BP stand for back propagation neural network; SVM stand for support vector machine; RF stand for random forest.
